# Phosphorus-independent role of FGF23 in erythropoiesis and iron homeostasis

**DOI:** 10.1371/journal.pone.0315228

**Published:** 2024-12-12

**Authors:** Min Young Park, Rafiou Agoro, Stanislovas S. Jankauskas, Carole Le Henaff, Despina Sitara

**Affiliations:** 1 Department of Molecular Pathobiology, New York University College of Dentistry, New York, NY, United States of America; 2 Department of Mammalian Genetics, The Jackson Laboratory, Bar Harbor, ME, United States of America; 3 Department of Medicine, Albert Einstein College of Medicine, New York, NY, United States of America; 4 Department of Medicine, Holman Division of Endocrinology, Diabetes and Metabolism, New York University Grossman School of Medicine, New York, NY, United States of America; University of Vermont, UNITED STATES OF AMERICA

## Abstract

A number of studies have reported an association between phosphorus, red blood cell (RBC) production, and iron metabolism. However, it is difficult to distinguish whether the effect of phosphorus is direct or through the actions of FGF23, and it is not clear whether phosphorus is positively or negatively associated with RBC production. In the present study, we investigated the effects of a) increased phosphorus load and b) phosphorus deficiency on erythropoiesis and iron metabolism in association with FGF23. Mice were fed either a 1.2% or 1.65% phosphorus diet and compared to mice fed a control diet containing 0.6% of phosphorus. Moreover, we used two mouse models of hypophosphatemia—induced either by dietary intervention in the form of a low phosphorus (LP) diet (0.02% of Pi) or genetically in a mouse model of X-linked hypophosphatemia (XLH)–that had opposite FGF23 levels. Phosphorus supplementation appropriately increased FGF23 levels leading to excretion of excess phosphorus and normalization of serum phosphorus levels. We also found that a phosphorus-rich diet results in inflammation-induced hypoferremia associated with reduced iron export leading to tissue iron overload. Moreover, high phosphorus intake results in ineffective erythropoiesis caused by decreased production (decreased RBCs, hemoglobin, hematocrit, and erythroid progenitors in the bone marrow) and increased destruction of RBCs, leading to anemia despite increased EPO secretion. These complications occur through the actions of elevated FGF23 in the presence of normophosphatemia. Our data also show that LP diet induces a decrease in the serum concentrations of phosphorus and FGF23, resulting in increased RBC counts, hemoglobin concentration, and hematocrit compared to mice fed normal diet. Moreover, serum iron and transferrin saturation were increased and positively correlated with serum ferritin, liver ferritin protein and mRNA expression in mice fed LP diet. However, hyp mice, the murine model of XLH, exhibit hypophosphatemia and high serum FGF23 levels, along with low number of circulating RBCs, hemoglobin, and hematocrit compared to wild-type mice. In the bone marrow, hyp mice showed reduced number of erythroid progenitors and formed significantly less BFU-E colonies compared to control mice. Serum iron levels and transferrin saturation were also decreased in hyp mice in comparison to control mice. Taken together, our data show that FGF23 acts independent of phosphorus levels to regulate erythropoiesis and iron homeostasis.

## Introduction

Phosphorus plays a critical role in several physiological processes including energy production, cellular respiration, and bone mineral metabolism, as an integral component of nucleic acids (DNA and RNA), cell membranes (forming phospholipids), high energy molecules (e.g. adenosine triphosphate (ATP)), and hydroxyapatite crystals. In the body, almost all phosphorus is combined with oxygen, forming phosphate. The majority of the body’s phosphate pool (about 85%) is stored in bone, while the rest is located primarily inside cells, where it is involved in energy production. Phosphate homeostasis is tightly regulated by 3 main hormones, parathyroid hormone (PTH), 1,25(OH)_2_D, and fibroblast growth factor 23 (FGF23), that control intestinal absorption, exchange with intracellular and bone storage pools, and renal excretion [1–3]. There are two main sources of phosphorus in the diet, organic phosphorus from natural foods (e.g. meat, fish, and dairy products), and inorganic phosphorus from food additives. While phosphate deficiency is very rare, hyperphosphatemia is a common finding in Western countries due to consumption of processed foods rich in phosphorus-based additives [4]. FGF23 is very sensitive to and positively correlated with dietary phosphorus levels [5]. When renal function is intact, phosphate surplus is excreted in the urine through the actions of FGF23 and PTH. However, when renal function is compromised, as it is in chronic kidney disease (CKD), phosphate excretion is impaired, leading to persistent hyperphosphatemia and continuous stimulation of FGF23.

Work from our lab demonstrated that increased FGF23 is a causative factor in the development of anemia, iron deficiency, and inflammation in mice with renal failure but also in mice with normal kidney function [6,7]. Importantly, we have shown that blocking FGF23 signaling stimulates red blood cell (RBC) production and rescues anemia, iron deficiency, and inflammation (chronic or acute) [6,7].

A number of studies have reported an association between phosphorus and red blood cells, however, it is not clear whether phosphorus is positively or negatively associated with RBC production [8–12]. Because of the interdependency between dietary phosphorus overload and FGF23, it is challenging to distinguish whether excess FGF23 and/or excess phosphorus directly contribute to anemia and iron deficiency.

Given the importance of phosphate in several biological processes, in the present study we investigated the effects of 1) increased dietary phosphorus intake and 2) phosphorus deficiency on erythropoiesis and iron metabolism in association with FGF23, using either dietary intervention in the form of high (1.2% Pi and 1.65% Pi) or low phosphorus (0.02% Pi) diet in mice with normal kidney function, or a mouse model of X-linked hypophosphatemia (XLH), the most common genetic disorder of phosphate homeostasis.

## Materials and methods

### Animals

C57BL/6J wild-type (WT) male mice 6 weeks of age were purchased from The Jackson Laboratory (Bar Harbor, ME, USA) and housed at New York University (NYU) College of Dentistry Animal Facility, where they were kept on a light/dark (12h/12h) cycle at 23°C, and received food (standard chow) and water *ad libitum*. After 1 week of acclimatization upon arrival, all groups of mice were fed control diet containing 0.6% inorganic phosphorus (Pi) (Adjusted phosphorus diet TD 98243, Harlan Teklad, Madison, WI) for 5 days. Then, each group of mice was switched to either control (0.6% Pi), high phosphorus (1.2% Pi, TD 02120 or 1.65% Pi, TD 86129), or low phosphorus (0.02% Pi, TD.86128) diet for 2 weeks. Male mice carrying a mutation in the *Phex* gene (hyp mice, a murine homolog of human XLH) and wild-type (WT) littermate mice were fed normal chow and analyzed at 8 weeks of age. At the end of the 2-week diet period (or at 8 weeks of age for hyp mice), mice were placed under deep anesthesia by isoflurane prior to blood drawing and sacrificed by isoflurane overdose followed by cervical dislocation. No animals were found dead during the study. All animal welfare considerations were taken, including efforts to minimize suffering and distress. Animals were monitored 3 times per week. Any animals exhibiting a profile indicative of pain or suffering (e.g., severely abnormal respiratory exchange ratio, little to no locomotor activity, body weight loss more than 15%, or insufficient food or water intake) were immediately removed from the study and humanely euthanized.

All animal procedures were conducted in strict accordance with protocols approved by the Institutional Animal Care and Use Committee (IACUC) at New York University (IA16-00648).

### Colony-forming unit assay

Cell suspensions were prepared from flushed bone marrow in 20% IMDM from mice fed different phosphorus diets (0.6%, 1.2%, and 1.65%). Aliquots were then plated in a semi-solid methylcellulose medium supplemented with recombinant cytokines for colony assays of murine cells (Methocult M3434; StemCell Technologies, Vancouver, BC, Canada). Cytokines included: IL-3 (4ng/10μl), stem cell factor (20ng/10μl), granulocyte macrophage-colony stimulating factor (2ng/10μl), and erythropoietin (2U/10μl). Bone marrow cells were plated at a concentration of 2 X 10^4^ and incubated in a humidified chamber at 37°C with 5% CO_2_. Colonies representing burst forming unit of erythroid cells (BFU-E), were scored between 8 and 12 days post-culture.

### Blood, serum, and tissue collection

Mice were immediately necropsied after euthanasia and blood was collected by cardiac puncture. Whole blood was collected in Microtainer^®^ Blood Collection Tubes with K_2_EDTA (Becton, Dickinson and Company, Franklin Lakes, NJ) and complete blood count was performed using the VetScan HM5 Hematology Analyzer (ABAXIS, Union City, CA). For serum, blood was obtained in separate Microtainer^®^ serum separator tube (Becton, Dickinson and Company, Franklin Lakes, NJ) and centrifuged at 1,200 x g for 10 min. Liver, spleen, kidney, intestine, bone marrow cells obtained from femora and tibiae, and bones (diaphysis) were collected, snap-frozen in liquid nitrogen, and stored at –80°C until further use.

### Serum measurements

Serum and urinary phosphorus and creatinine, and serum calcium levels were determined by colorimetric measurements using the Stanbio Phosphorus Liqui-UV^®^, Stanbio Direct Creatinine LiquiColor^®^ Test reagents, and Stanbio total calcium LiquiColor^®^, respectively (Stanbio Laboratory, Boerne, TX). Serum FGF23 levels were measured using mouse FGF23 Intact and C-terminal ELISA assays (Quidel Corporation/Immutopics International, San Clemente, CA, USA). Serum iron and transferrin saturation were measured using the Iron-TIBC kit from Pointe Scientific (Canton, MI, USA). Serum erythropoietin (EPO) and parathyroid hormone (PTH) levels were measured using the Rat/Mouse EPO Quantikine (R&D Systems, Minneapolis, MN, USA), and MicroVue Mouse PTH 1–84 (Quidel Corporation/Immutopics International, San Clemente, CA, USA) ELISA assays, respectively. Serum hepcidin was measured using the Hepcidin Murine-Compete ELISA kit (Intrinsic LifeSciences, La Jolla, CA, USA).

### Tissue iron measurement

Hepatic, renal, and splenic iron content was measured by the Ferrozine colorimetric method, as described previously [13,14].

### RNA isolation, reverse transcription, and real-time quantitative PCR analysis

Total RNA was extracted from kidneys, liver, diaphysis, spleen, and bone marrow using Trizol (Ambion; Life Technologies, Carlsbad, CA, USA) according to the manufacturer’s protocol (Molecular Research Center, Cincinnati, OH, USA). cDNA was synthesized using the High Capacity cDNA Reverse Transcription Kit, as described by the manufacturer (Applied Biosystems; Thermo Fisher Scientific, Waltham, MA, USA), and amplified by quantitative PCR (qPCR) using the PerfeCTa SYBR Green SuperMix (Quanta Biosciences, Gaithersburg,MD, USA). All primers used in this study are listed in **Table 1**. mRNA levels were normalized to the housekeeping gene (*Hprt*) in the same RT sample. The relative transcript expression of a gene is given as Δ*C*t = *C*t_target_−*C*t_reference_. The fold change in gene expression, as compared to control mice, was determined as 2^−ΔΔ*C*t^ values (ΔΔ*C*t = Δ*C*t_treated_−Δ*C*t_control_).

**Table 1:** Primer sequences.

Gene	Primer	Sequence (5’ - 3’)
*Klotho*	Forward primer	AAA TGG CTG GTT TGT CTC GGG AAC
	Reverse primer	TAT GCC ACT CGA AAC CGT CCA TGA
*NaPi2a*	Forward primer	GTG CCT CTG ATG CTG GCT TTC
	Reverse primer	CTG GAA CTC TGC ACC AGA ACT
*NaPi2c*	Forward primer	CTC ACC ATA CAT GCA GAG CTA GGA
	Reverse primer	TGC ATT TCT CAG ACT CCG GT
*Cyp27b1*	Forward primer	GCT CGC CTC CAG AGT TTT TC
	Reverse primer	AAA CTG TGC GAA GTG TCC CA
*Cyp24a1*	Forward primer	ACG TCA CCT CCT TAC CTG GA
	Reverse primer	GAT GCA CCG AGT CGA AGG AG
*Tnfα*	Forward primer	AAG GGA GAG TGG TCA GGT TGC C
	Reverse primer	CCT CAG GGA AGA GTC TGG AAA GG
*IL-6*	Forward primer	ATC CAG TTG CCT TCT TGG GAC TGA
	Reverse primer	TAA GCC TCC GAC TTG TGA AGT GGT
*Hepcidin (Hamp)*	Forward primer	CAC CAC CTA TCT CCA TCA ACA G
	Reverse primer	GTT GGT GTC TCT CTT CCT TCT C
*Epo*	Forward primer	TCT ACG TAG CCT CAC TTC ACT
	Reverse primer	ACC CGG AAG AGC TTG CAG AAA
*EpoR*	Forward primer	GGG CTG CAT GGA CAA ACT
	Reverse primer	GCC GCT TTG CTC TCA AAC TT
*Hif2α*	Forward primer	GGG AAC ACT ACA CCC AGT GC
	Reverse primer	TCT TCA AGG GAT TCT CCA AGG
*Erfe*	Forward primer	TCT GGG ACC ATC CAG CTT AC
	Reverse primer	ATC GTG CTT ACC CCA GAC AC
*Hmox*	Forward primer	GCC GAG AAT GCT GAG TTC ATG
	Reverse primer	TGG TAC AAG GAA GCC ATC ACC
*Hprt*	Forward primer	AAG CCT AAG ATG AGC GCA AG
	Reverse primer	TTA CTA GGC AGA TGG CCA CA

### Isolation and assessment of bone marrow (BM) cells by flow cytometry analysis

Bone marrow was isolated from dissected tibiae and femora from mice fed different phosphate diets and hyp (and control) mice, as previously described. Briefly, BM cell suspensions were prepared by flushing bone marrow in Iscove’s modified Dulbecco’s medium (IMDM) supplemented with 20% fetal bovine serum (20% IMDM) through a 26G Becton Dickinson needle. BM cells were dispersed by manual agitation then filtered to remove foreign particles. Flow cytometry analysis for BM cells was carried out in a BD FACSort flow cytometer equipped with 488 argon lasers (BD Biosciences). For immunostaining, cells were washed and resuspended in 1x PBS containing 0.1% bovine serum albumin. Mouse FcR was blocked before staining using CD16 / 32 antibody to reduce nonspecific binding. After the addition of antibodies, cells were incubated for 40 min on ice. Labeled cells were then washed with 1x PBS and analyzed by flow cytometry. Appropriate isotype controls were kept for each set. Forward- and side- scatter patterns were gated excluding the debris. A total of 20,000 events were collected and analyzed by FlowJo 7.6.5 software (FlowJo, Ashland, OR, USA). Erythroid lineage was assessed using Ter119-APC/CD71-PE/CD44-FITC markers (BD Pharmingen, San Jose, CA, USA) combined with the forward-scatter properties [15,16].

### Colony-forming unit assay

Cell suspensions were prepared from flushed bone marrow in 20% IMDM from mice fed different phosphorus diets (0.6%, 1.2%, and 1.65%). Aliquots were then plated in a semi-solid methylcellulose medium supplemented with recombinant cytokines for colony assays of murine cells (Methocult M3434; StemCell Technologies, Vancouver, BC, Canada). Cytokines included: IL-3 (4ng/10μl), stem cell factor (20ng/10μl), granulocyte macrophage-colony stimulating factor (2ng/10μl), and erythropoietin (2U/10μl). Bone marrow cells were plated at a concentration of 2 X 10^4^ and incubated in a humidified chamber at 37°C with 5% CO_2_. Colonies representing burst forming unit of erythroid cells (BFU-E), were scored between 8 and 12 days post-culture.

### Western blotting

Snap frozen samples of spleen, liver, and kidney were homogenized in an ice-cold RIPA buffer (Sigma-Aldrich, St. Louis, MO, USA) and protein was extracted, electrophoresed, transferred to nitrocellulose membrane, and incubated with anti-Ferroportin rabbit antibody (#MTP11-S, Alpha Diagnostic Intl Inc., San Antonio, TX, USA) or anti-Actin goat antibody (sc-1616, Santa Cruz Biotechnology Inc., Dallas, TX, USA), as described previously [7].

### Statistics

All data were analyzed using GraphPad Prism version 8.0 for Windows (GraphPad Software, San Diego, CA, USA). For correlation curve, we applied simple linear and simple logistic regression. For validation of normal distribution, Shapiro-Wilk test was carried out and for homogeneity of variance, we Brown-Forsythe test was performed. For samples with normal distribution and equal variance, one-way analysis of variance (ANOVA) was performed followed by Dunnett’s multiple comparison test for multiple comparisons. When the samples were in normal distribution but not in homogeneity of variance, Welch’s ANOVA was performed. When the data were not normally distributed, non-parametric (Kruskal-Wallis) test followed by Dunn test was performed. All data were expressed at mean ± SD. P values less than 0.05 were considered significant.

## Results

### Regulation of phosphate, calcium, and vitamin D metabolism by FGF23 in response to high phosphorus diet

**Fig 1:**
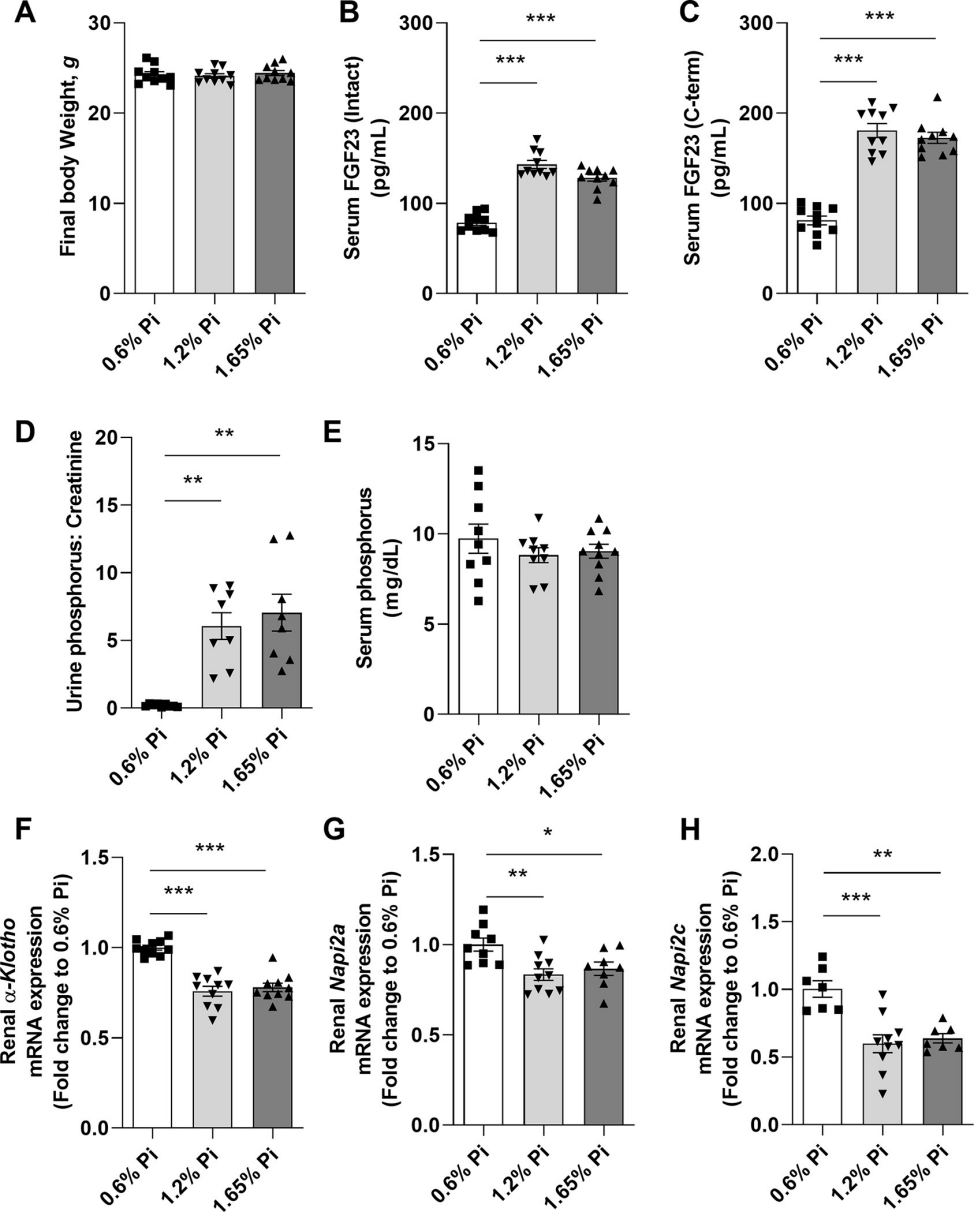
Phosphate regulation in response to high phosphorus diet intake. C57BL/6J male mice were fed a diet containing 0.6% inorganic phosphorus (Pi), 1.2% Pi, or 1.65% Pi for 2 weeks. **(A)** Body weight after 2 weeks of phosphorus supplementation. **(B-C)** Serum concentration of **(B)** intact and **(C)** C-terminal FGF23 measured by ELISA. **(D-E)** Phosphorus levels measured in **(D)** urine as ratio of phosphorus to creatinine and **(E)** serum. **(F-H)** Quantitative real-time RT-PCR for renal **(F)** *α-Klotho*, **(G)** *Napi2a*, and **(H)** *Napi2c* expression. Data are expressed as fold change (2^-ΔΔCt^) relative to housekeeping gene *Hprt*. Samples were measured in duplicates. Data are represented as mean ± SD. All data were analyzed for normality with Shapiro-Wilk test and homogeneity of variance by Brown-Forsythe test. For samples with normal distribution, one-way ANOVA was performed compared to 0.6% Pi with Dunnett’s multiple comparison test (B, C, E, F, G, H). When the samples were in normal distribution but not in homogeneity of variance, Welch’s ANOVA was performed (D). The samples not in normal distribution were analyzed with non-parametric Kruskal-Wallis test (A). (n = 7–10 per group). *P < 0.05, **P < 0.01, ***P < 0.001 compared to 0.6% Pi.

In the present study, we fed wild-type (WT) C57BL6/J mice either a moderately high (1.2% Pi) or an acutely high (1.65% Pi) phosphorus diet for 2 weeks and compared them to mice fed normal phosphorus diet (0.6% Pi; control diet). Our data show that intake of high dietary phosphorus did not cause any change in body weight in mice fed either 1.2% Pi or 1.65% Pi compared with mice fed 0.6% Pi diet (**Fig 1A**). To validate the effect of high phosphorus intake in the regulation of phosphate homeostasis, we measured phosphorus levels in serum and urine, as well as circulating intact and C-terminal FGF23 levels. Both intact (**Fig 1B**) and C-terminal FGF23 levels (**Fig 1C**) were markedly elevated in response to excess phosphorus intake in comparison to mice fed 0.6% Pi. The percentage of intact to C-terminal FGF23 is shown in **S1A Fig**. Consistent with its role as a phosphaturic hormone, elevated serum FGF23 led to a significant increase in urinary phosphate excretion (**Fig 1D**), resulting in normal serum phosphate levels (**Fig 1E**). FGF23 exerts its phosphaturic action by binding to the FGFR-Klotho complex in the kidney to downregulate the sodium-phosphate transporters NaPi2a/c and induce phosphate excretion [17,18]. As expected, high FGF23 led to a significant decrease in renal *Klotho* (**Fig 1F**), *NaPi2a* (**Fig 1G**), and *NaPi2c* (**Fig 1H**) expression in mice fed 1.2% Pi and 1.65% Pi compared to mice fed 0.6% Pi. Taken together, our data show that a 2-week dietary phosphorus overload raised serum FGF23, which, in turn, maintained serum phosphorus homeostasis by promoting phosphate excretion via its phosphaturic action.

**Fig 2:**
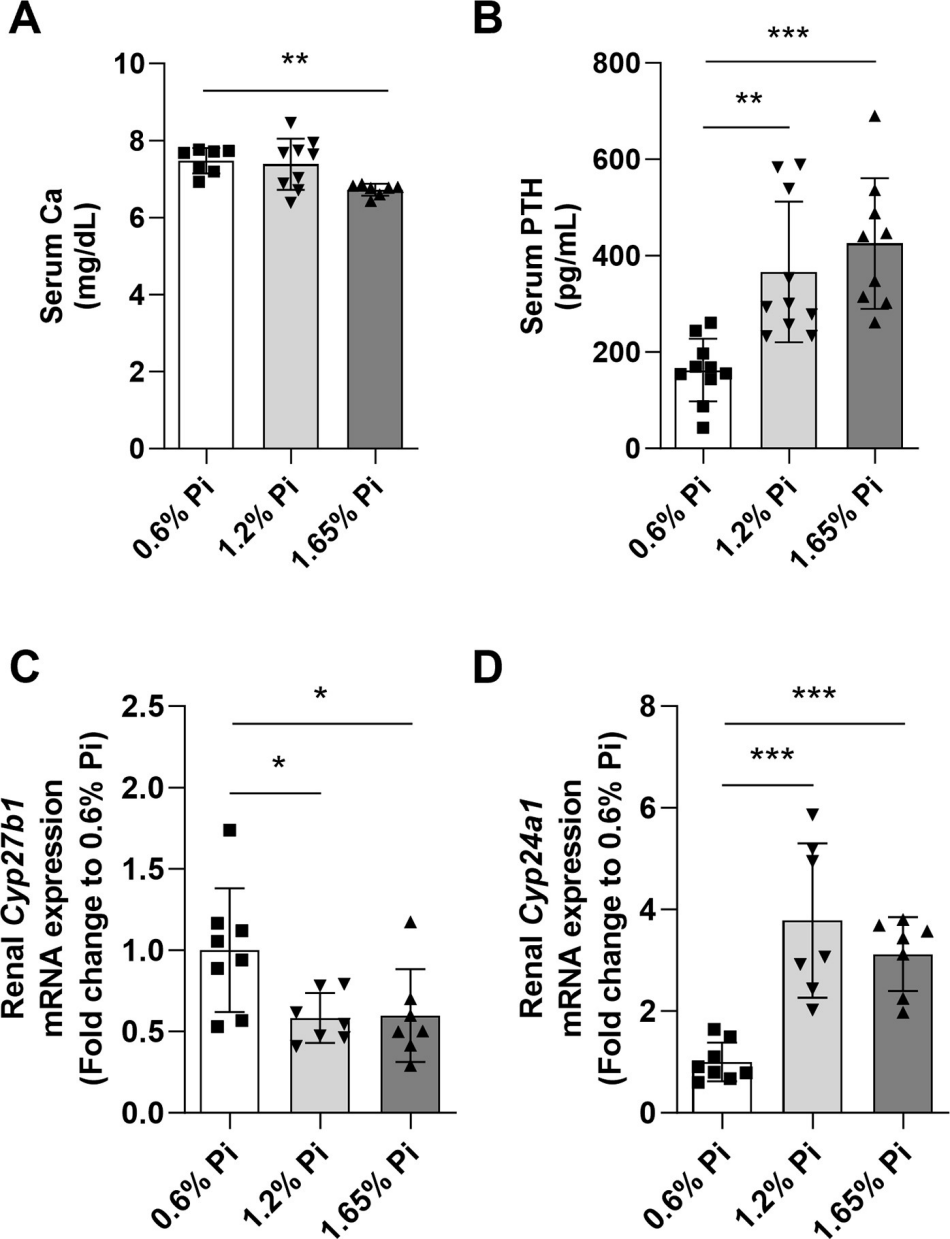
Regulation of calcium homeostasis following phosphorus supplementation. **(A-B)** Serum levels of **(A)** calcium (Ca), and **(B)** PTH measured after 2 weeks of different dietary phosphorus intake. **(C-D)** Quantitative real-time RT-PCR for renal **(C)** *cyp27b1* and **(D)** *cyp24a1* expression. Data are expressed as fold change (2^-ΔΔCt^) relative to housekeeping gene *Hprt*. Samples were measured in duplicates. Data are represented as mean ± SD. All data were analyzed for normality with Shapiro-Wilk test and homogeneity of variance by Brown-Forsythe test. For samples with normal distribution with equal variance, one-way ANOVA with Dunnett’s multiple comparison test was performed (B, C). Samples with unequal variance were analyzed with Welch’s ANOVA (A, D). (n = 7–10 per group). *P < 0.05, **P < 0.01, ***P < 0.001 compared to 0.6% Pi.

It has also been shown that high phosphate diets result in decreased intestinal calcium absorption, reducing serum calcium concentration, and stimulating PTH secretion, and these immediate changes are independent of serum phosphorus levels [19–21]. In agreement with these studies, our data show that serum calcium levels decreased (**Fig 2A**), whereas circulating PTH levels significantly increased (**Fig 2B**) in mice fed high Pi compared to mice fed normal Pi diet. Interestingly, PTH increased in 1.2% phosphorus diet (**Fig 2B**) even though there were not any detectable changes in serum calcium levels (**Fig 2A**). It is possible that fluctuations in serum Ca caused by high dietary phosphate (1.2%) were immediately corrected by PTH, whereas intake of 1.65% of phosphorus had a bigger effect on calcium levels for PTH to correct and normalize. Moreover, high phosphorus intake, directly and through Pi-induced increase in circulating FGF23, has been shown to inhibit 1,25(OH)_2_D production in the kidney by suppressing renal 1α-hydroxylase (*Cyp27b1*) and stimulating 24-hydroxylase (*Cyp24a1*) mRNA abundance [5,22,23]. In our studies we confirmed a decrease in *Cyp27b1* (**Fig 2C**) and increase in *Cyp24a1* (**Fig 2D**) renal expression in response to high phosphate diet feeding. Together, our data demonstrate that dietary intake of high phosphorus for 2 weeks does not result in hyperphosphatemia but elevates circulating FGF23, which aids the kidney to appropriately handle the phosphate load from the diet by inducing phosphate excretion (via downregulation of the renal phosphate transporters NaPi2a/c) and reducing phosphate absorption in the intestine (via decrease in 1,25(OH)_2_D synthesis). Decreased 1,25(OH)_2_D secretion also results in reduced intestinal calcium absorption, leading to hypocalcemia and increase in PTH levels.

### Effect of high dietary phosphorus intake on iron homeostasis

Dietary phosphate overload has been reported to directly induce systemic inflammation in CKD [24]. Inflammation induces secretion of the hepatic hormone hepcidin, the central regulator of iron homeostasis [25,26]. Iron is tightly regulated between duodenal iron absorption and macrophage iron recycling. In the intestine, hepcidin binds to ferroportin (FPN), the only known mammalian iron exporter, and causes its internalization and degradation [27], limiting iron efflux from reticulo-endothelial macrophages, hepatocytes, and duodenal enterocytes to the circulation [28].

A relationship between iron and phosphate metabolism has been reported in several studies mainly with regards to low phosphate levels. Animal studies have shown that iron deficiency induced by a low-iron diet stimulates *Fgf23* transcription in bone [29], and suppresses intestinal phosphate absorption by downregulating the phosphate transporter *NaPi2b* in the duodenum [30], leading to hypophosphatemia [31]. Moreover, certain iron formulations and intravenous iron supplementation can increase circulating FGF23 levels by inhibiting FGF23 cleavage [32]. Conversely, iron replacement decreases FGF23 production [32,33]. Short-term use of the phosphate binder ferric citrate was shown to ameliorate iron deficiency anemia and hyperphosphatemia, and reduce FGF23 levels in CKD patients [34–36]. However, the effects of dietary phosphate supplementation on iron metabolism are not fully understood.

**Fig 3:**
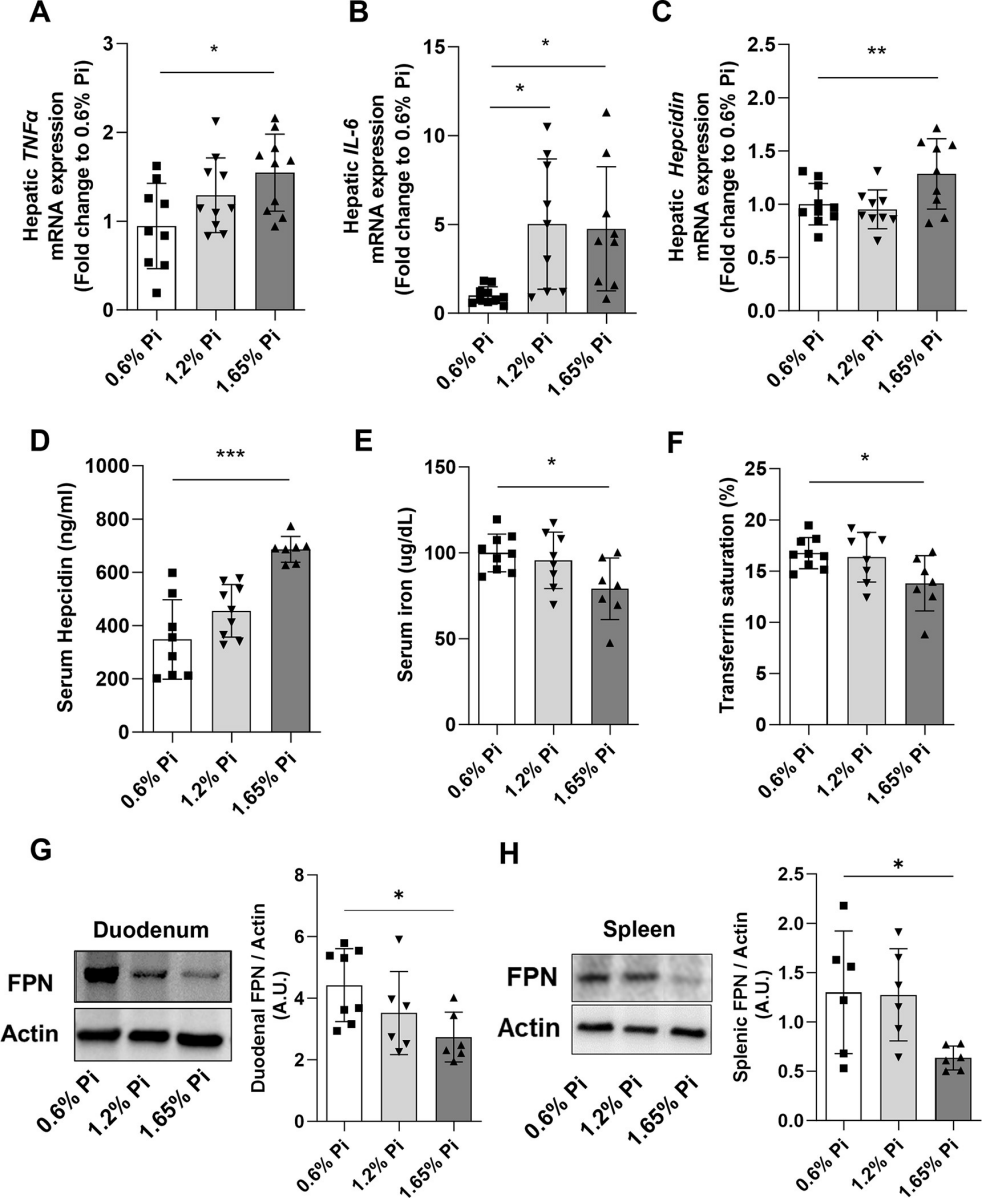
Effect of dietary phosphorus overload on inflammation and iron homeostasis. C57BL/6J male mice were fed a diet containing 0.6% inorganic phosphorus (Pi), 1.2% Pi, or 1.65% Pi for 2 weeks. Serum and tissue samples were collected at the end of the experiment. **(A-C)** Quantitative real-time RT-PCR for hepatic expression of **(A)** *TNFα*, **(B)** *IL-6*, and **(C)** *hepcidin*. **(D-F)** Serum levels of **(D)** hepcidin, **(E)** iron, and **(F)** transferrin saturation. **(G-H)** Protein expression of Ferroportin (FPN) in **(G)** duodenum and **(H)** spleen, shown in representative image of western blot (left) and quantification (right) in each panel. Proteins extracted from each tissue were analyzed by Western blot assay. Abundance of FPN protein was normalized with Actin. Data are represented as mean ± SD. All data were analyzed for normality with Shapiro-Wilk test and homogeneity of variance by Brown-Forsythe test. When the samples showed normal distribution, one-way ANOVA was performed (A, C, E, F, G). When the samples did not show homogeneity of variance, logarithmic transformation of data was performed and equivalence of variance was confirmed once again prior to one-way ANOVA (B, H). The samples not in homogeneity of variance (normal distribution) were analyzed with non-parametric Kruskal-Wallis test (D). (n = 6–8 per group). *P < 0.05, **P < 0.01, ***P < 0.001 compared to 0.6% Pi.

**Fig 4:**
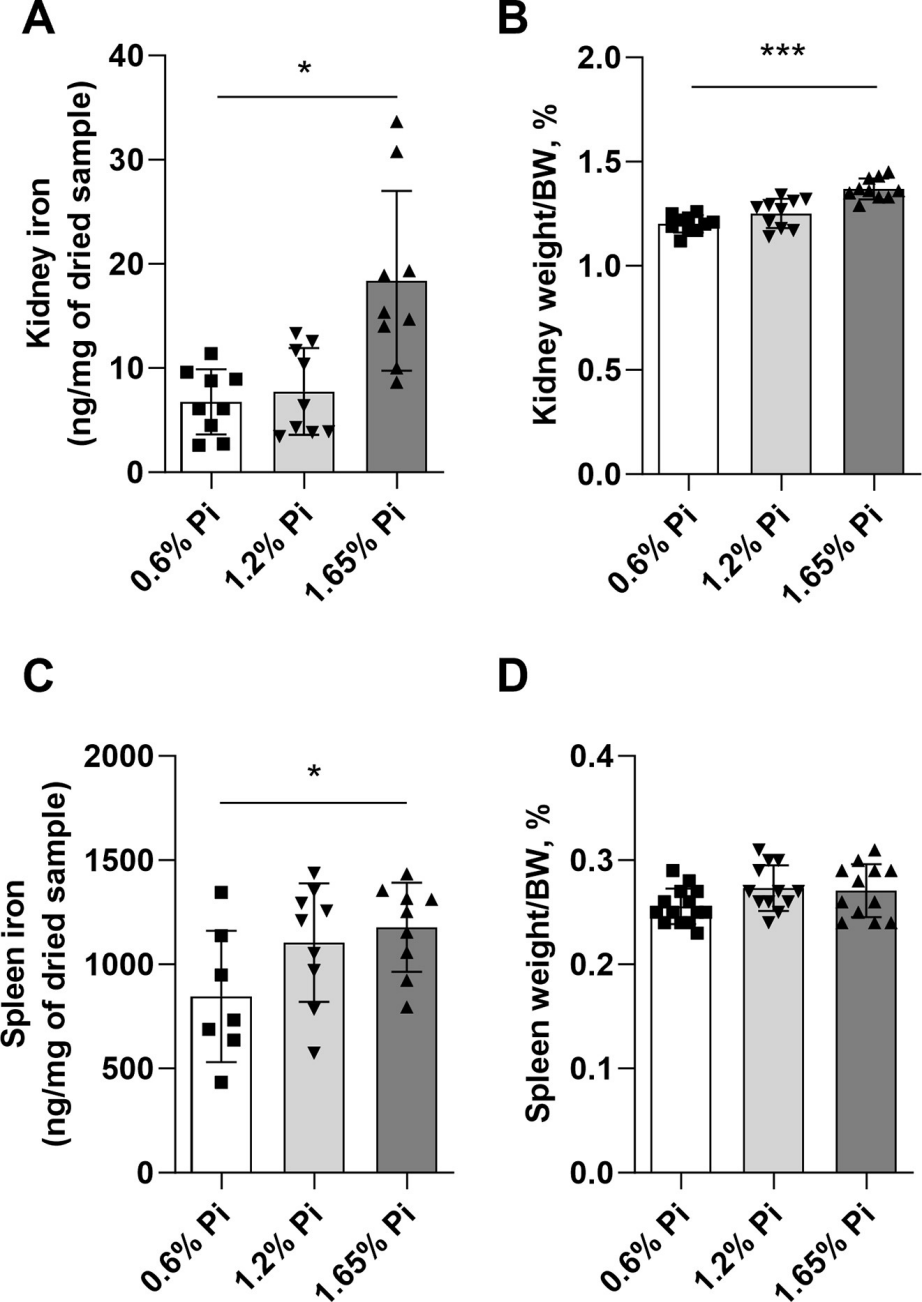
Tissue iron content following high phosphorus intake. Tissue iron content was measured after 2 weeks of phosphorus overload and normalized to weight (mg) of dried tissue sample. Tissues were weighed at collection and their weight was normalized to total body weight of the animal and represented as percentage. **(A)** Kidney iron content, **(B)** kidney weight, **(C)** spleen iron content, and **(D)** spleen weight. Data are represented as mean ± SD. All data were analyzed for normality with Shapiro-Wilk test and homogeneity of variance by Brown-Forsythe test. Samples were in normal distribution with equal variance, and one-way ANOVA with Dunnett’s multiple comparison test was performed (A-D). (n = 7–10 per group). *P < 0.05, **P < 0.01, ***P < 0.001 compared to 0.6% Pi.

In the present study, we evaluated the effect of high phosphate diet on inflammation and iron homeostasis. Our data show that pro-inflammatory cytokines such as *TNFα* and *IL-6* were significantly upregulated in the liver of mice fed 1.65% Pi diet for 2 weeks (**Fig 3A and 3B**). Consistent with elevated *TNFα* and *IL-6* levels, hepatic hepcidin expression was also upregulated (**Fig 3C**) leading to increased secretion of hepcidin in the circulation (**Fig 3D**) in mice fed 1.65% Pi compared to mice fed 0.6% (control) Pi diet. The increase in hepcidin resulted in significant reduction in circulating iron and transferrin saturation levels (**Fig 3E and 3F**) in the high Pi diet (1.65% Pi) group. Moreover, this drop in serum iron levels was accompanied by a significant decrease in ferroportin protein expression in duodenum (**Figs 3G** and **S2A**) and spleen (**Figs 3H** and **S2B**), resulting in iron retention in kidney (**Fig 4A**) and spleen (**Fig 4C**), which ultimately led to increased weight of these organs (**Fig 4B and 4D**) in mice fed high Pi (1.65% Pi) diet. Taken together, these data suggest that increased intake of dietary phosphate induces inflammation and hepcidin production, resulting in hypoferremia associated with iron overload in spleen and kidneys.

### High phosphorus diet results in anemia and ineffective erythropoiesis

Iron is an integral component of hemoglobin and essential for red blood cell production. Several studies have reported that circulating iron restriction caused by inflammation-induced hepcidin activation promotes ineffective erythropoiesis and results in anemia of inflammation, a form of anemia commonly associated with chronic inflammatory conditions such as cancer and CKD [37–41]. Moreover, *in vitro* treatment with IFN-α or IFN-γ suppressed growth of erythroid colony-forming units (CFU-E), a precursor of erythrocytes [42,43]. There are only a few reports that link phosphorus to erythropoiesis. A couple of studies have shown that profound hypophosphatemia is associated with hemolytic anemia, which is corrected after parenteral Pi administration [44,45]. It has also been reported that phosphate deficiency may decrease the life span of red blood cells by negatively affecting the rate of erythrocyte glycolysis, which is critical for cellular integrity [8,46,47]. Furthermore, an association between hyperphosphatemia and anemia has been described in renal failure and also in individuals with normal kidney function [12,48]. However, the role of increased FGF23 levels by high phosphorus in these individuals was not taken into consideration in these studies.

**Fig 5:**
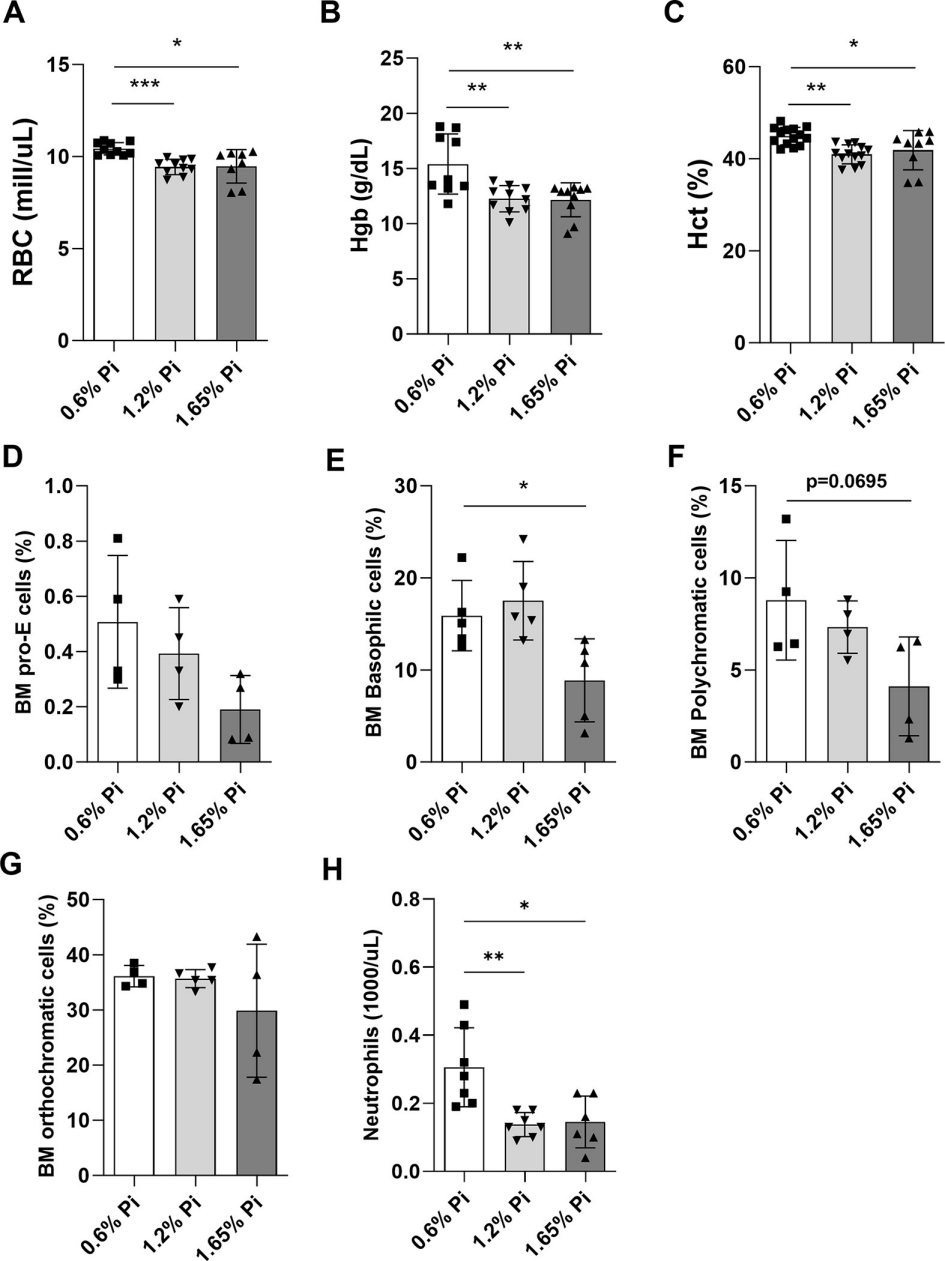
Effect of phosphorus overload on circulating red blood cell parameters and bone marrow erythroid progenitor cells. C57BL/6J male mice were fed a diet containing 0.6% inorganic phosphorus (Pi), 1.2% Pi, or 1.65% Pi for 2 weeks. Whole blood was collected and analyzed by complete blood count (CBC) for circulating red blood cell parameters (A-C) and FACS for bone marrow erythroid progenitor cells (D-G). **(A)** Red Blood Cells (RBCs), **(B)** Hemoglobin (Hgb), **(C)** Hematocrit (Hct). (n = 7–10 per group). **(D-G)** Bone marrow cells were collected from femur and tibia and further quantified for erythroid progenitor cell populations by FACS analysis for **(D)** proerythroblasts (pro-E), **(E)** basophilic, **(F)** polychromatic, and **(G)** orthochromatic cells (n = 4 per group). **(H)** Neutrophil counts obtained from CBC (n = 6–7 per group). Data are represented as mean ± SD. All data were analyzed for normality with Shapiro-Wilk test and homogeneity of variance by Brown-Forsythe test. For samples with normal distribution with equal variance, one-way ANOVA with Dunnett’s multiple comparison test was performed (B, C, D, E, F). Samples with normal distribution and unequal variance were analyzed with Welch’s ANOVA (G, H). Samples not in normal distribution were analyzed with non-parametric Kruskal-Wallis test (A). *P < 0.05, **P < 0.01, ***P < 0.001 compared to 0.6% Pi.

In our study we evaluated the effect of dietary Pi supplementation on red blood cell production and erythropoiesis. Our data show that mice fed 1.65% Pi diet for 2 weeks developed anemia and had significantly lower circulating RBCs (**Fig 5A**), hemoglobin concentration (**Fig 5B**), and hematocrit (**Fig 5C**). Moreover, we analyzed bone marrow erythroblasts by flow cytometry to separate cells at distinct stages of erythroid differentiation: pro-erythroblasts, basophilic erythroblasts, polychromatic erythroblasts, and orthochromatic erythroblasts. We found that all four populations of erythroid precursors were decreased in the bone marrow of mice fed 1.65% Pi compared to mice fed 0.6% (control) Pi diet (**Fig 5D–5G**).

**Fig 6:**
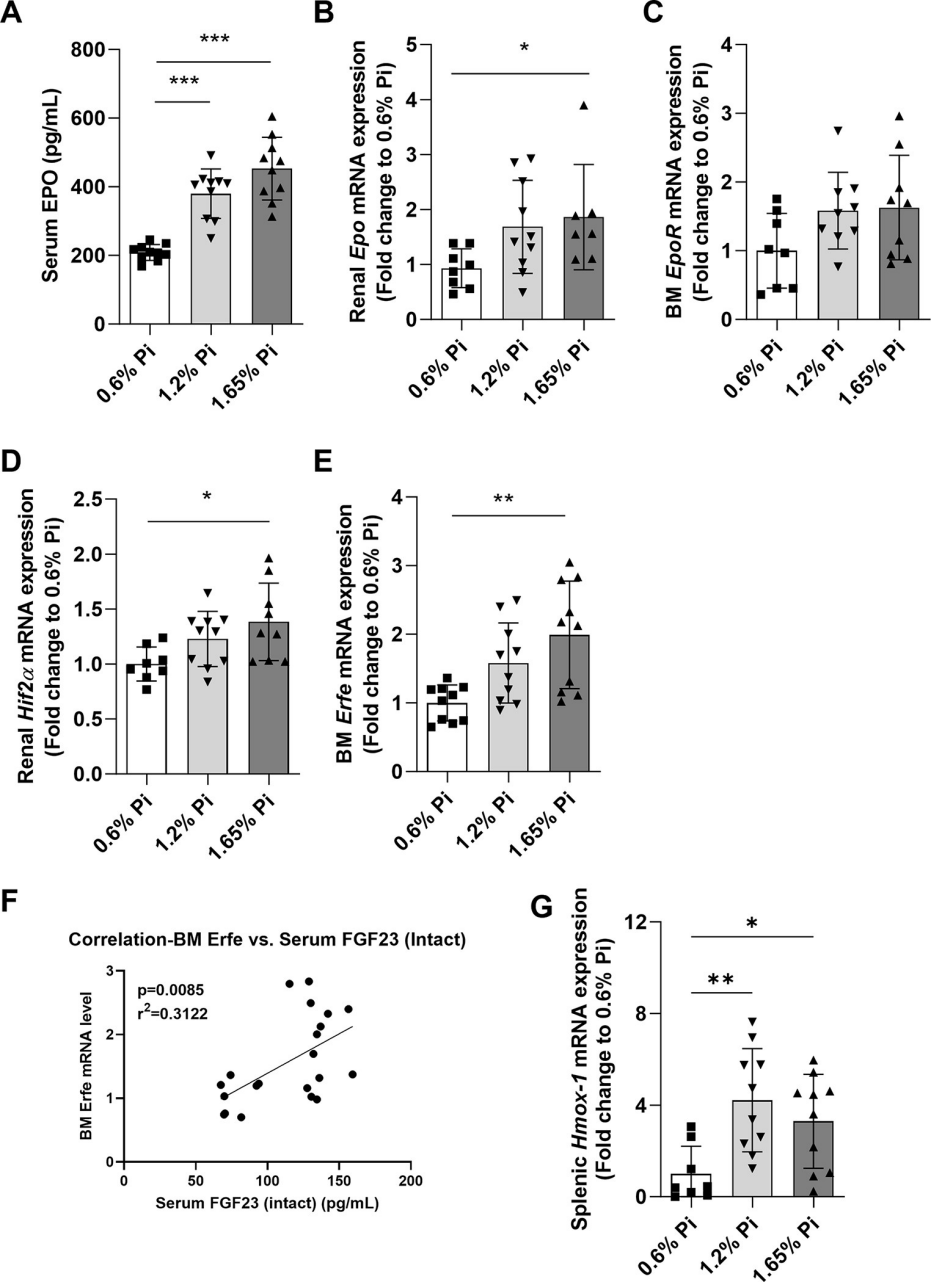
Activation of hypoxia inducible factors and Epo signaling pathway by phosphorus supplementation. C57BL/6J male mice were fed a diet containing 0.6% inorganic phosphorus (Pi), 1.2% Pi, or 1.65% Pi for 2 weeks. **(A)** Serum erythropoietin (EPO) levels measured by ELISA. **(B-E)** Quantitative real-time RT-PCR for expression of **(B)** renal *Epo*, **(C)** bone marrow Epo receptor (EpoR), **(D)** renal *Hif-2α*, and **(E)** bone marrow *erythroferrone* (*Erfe*). **(F)** Regression curve for correlation between bone marrow *Erfe* expression levels and serum FGF23 levels. **(G)** Quantitative real-time RT-PCR for splenic expression of *heme oxygenase-1* (*Hmox-1*). Data are expressed as fold change (2^-ΔΔCt^) relative to housekeeping gene *Hprt*. Data are represented as mean ± SD. All data were analyzed for normality with Shapiro-Wilk test and homogeneity of variance by Brown-Forsythe test. For samples with normal distribution and equal variance, one-way ANOVA with Dunnett’s multiple comparison test was performed (C, D, G). Samples with unequal variance were analyzed with Welch’s ANOVA (A, B, E). For the correlation curve, we applied simple linear and simple logistic regression (F). (n = 7–10 per group). *P < 0.05, **P < 0.01, ***P < 0.001.

The production of RBCs in the bone marrow is controlled by erythropoietin (EPO), a hormone mainly produced and secreted by the kidneys in response to hypoxia and anemia, which binds to its receptor (EPOR) on the surface of erythroid progenitors in the bone marrow. Thus, we further investigated the effect of dietary Pi overload on renal expression and circulating levels of EPO. Our data show that in response to decreased RBC production, serum EPO concentration (**Fig 6A**) and renal *Epo* expression (**Fig 6B**) were markedly increased in the high Pi diet (1.65% Pi) group compared to the control diet group (0.6% Pi). Moreover, *EpoR* and the hypoxia-inducible transcription factor (HIF) *Hif2*, a major stimulus of EPO, were upregulated in the bone marrow (**Fig 6C**) and kidney (**Fig 6D**), respectively, in mice fed 1.65% Pi diet, although *EpoR* upregulation did not reach statistical significance. Increased EPO production by the kidney increases the synthesis of the hormone erythroferrone (Erfe) in bone marrow erythroblasts, which acts on hepatocytes to suppress hepcidin synthesis, thereby promoting membrane ferroportin stabilization and mobilizing iron from tissues to the circulation. Indeed, *Erfe* expression was upregulated in the bone marrow of mice fed 1.65% Pi diet compared to the control diet group (**Fig 6E**). In addition, we found a significant positive correlation between *Erfe* and serum FGF23 levels (**Fig 6F**). To better understand the tissue (kidney and spleen) iron overload and presence of anemia in mice fed 1.65% Pi diet despite increased EPO secretion, we assessed the expression of heme oxygenase-1 (Hmox-1), an essential enzyme for iron recycling. High levels of Hmox-1 are detected in tissues that degrade aged red blood cells such as the spleen and liver [49]. Accordingly, spleen *Hmox-1* was upregulated in the high Pi diet (1.65% Pi) group compared to the control diet group (0.6% Pi) (**Fig 6G**). Taken together, our data suggest that a 2-week phosphate overload results in ineffective erythropoiesis caused by decreased production of red blood cells in the bone marrow as well as increased destruction of red blood cells in the spleen, leading to anemia that cannot be corrected by increased EPO secretion.

### Effect of low phosphorus diet on erythropoiesis and iron homeostasis

**Fig 7:**
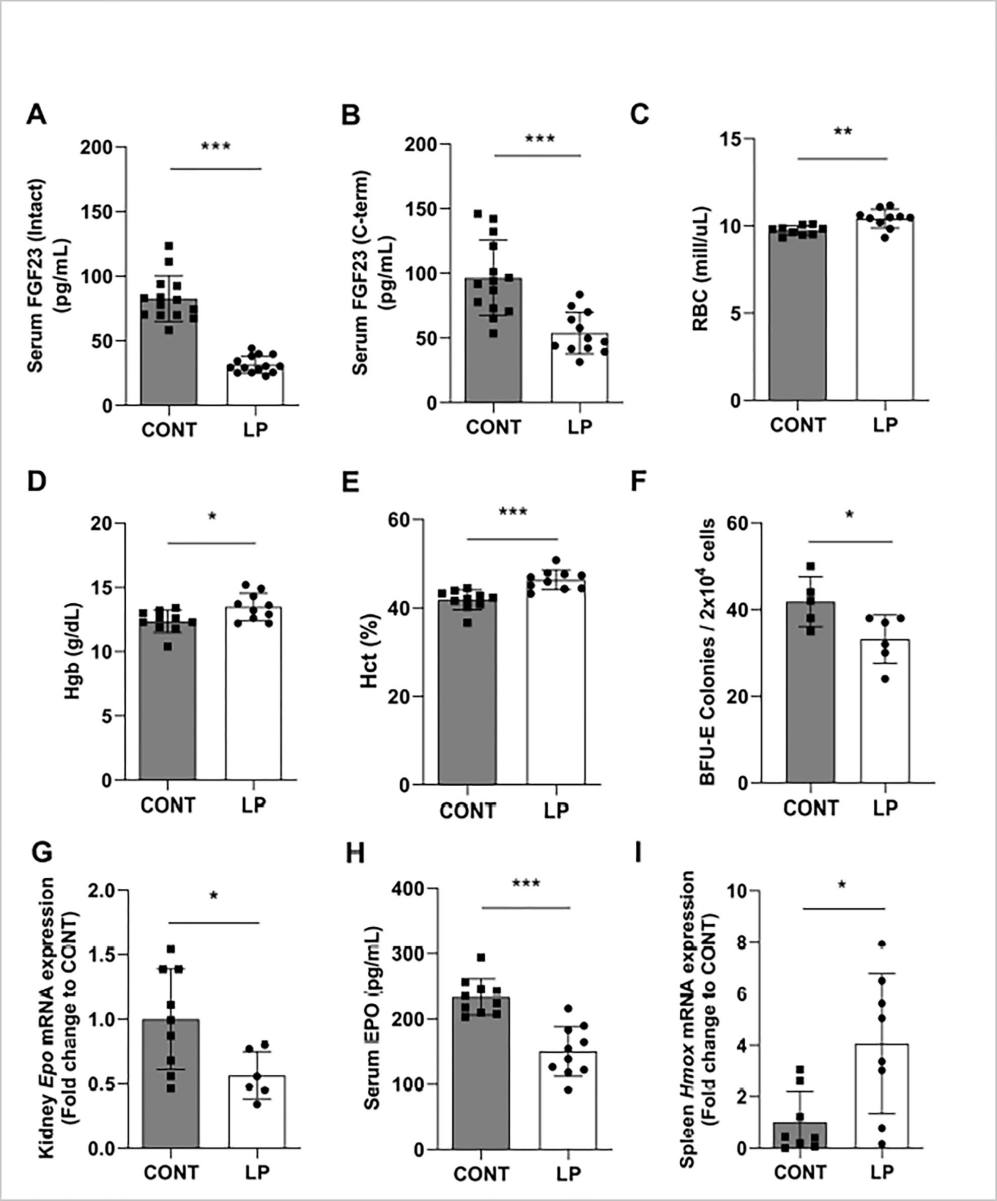
Low phosphorus diet increases red blood cell production through suppression of FGF23. Eight-week old C57BL/6J male mice were fed a diet containing 0.02% inorganic phosphorus (LP) for 2 weeks and compared to C57BL/6J male mice fed normal phosphorus diet (0.6% Pi; CONT). **(A-B)** Serum concentration of **(A)** intact FGF23, and **(B)** C-terminal FGF23 after 2 weeks of phosphorus restriction. **(C-E)** Circulating red blood cell parameters. **(C)** Red Blood Cells (RBCs), **(D)** Hemoglobin (Hgb), **(E)** Hematocrit (Hct). **(F)** Colony forming unit assay. Bone marrow cells were isolated from femora and tibiae, cultured for 12 days on methylcellulose medium, and counted for erythroid progenitor cells, burst forming unit-erythroid (BFU-E). **(G)** Quantitative real-time RT-PCR for renal *Epo* expression. Data are expressed as fold change (2^-ΔΔCt^) relative to housekeeping gene *Hprt*. **(H)** Serum concentration of EPO. Samples were measured in duplicates. **(I)** Quantitative real-time RT-PCR for splenic *Hmox* expression. Data are expressed as fold change (2^-ΔΔCt^) relative to housekeeping gene *Hprt*. Data are represented as mean ± SD. (n = 7–10 per group for A, B, C, D, E, F, H, and I; n = 5–6 per group for G). All data were analyzed for normality with Shapiro-Wilk test and homogeneity of variance by F test. For samples with normal distribution, unpaired t test was performed compared to CONT (B, C, D, E, F, G, H). When the samples were in normal distribution but not in homogeneity of variance, the data were analyzed by Welch’s t test (A, I). *P < 0.05, **P < 0.01, ***P < 0.001 compared to CONT (control diet).

Acquired phosphorus deficiency was achieved in wild-type C57BL/6J mice by feeding a low phosphorus diet (0.02% Pi) for 2 weeks and compared to C57BL/6J mice fed normal (control) phosphorus diet (0.6% Pi). As expected, the serum phosphorus concentration was significantly reduced after 2 weeks of low dietary phosphorus (LP) intake (**S3A Fig**) and correlated with a significant reduction in circulating FGF23 levels, both intact and C-terminal (**Fig 7A and 7B**), in agreement with published data [5,50–52]. The percentage of intact to C-terminal FGF23 is shown in **S1B Fig**. Restriction of dietary phosphorus in normal healthy individuals induces an increase in vitamin D production in the kidney by stimulating *1α-hydroxylase* (*Cyp27b1*) renal mRNA abundance and resulting in elevated circulating levels of 1,25(OH)_2_D [5,22]. In turn, 1,25(OH)_2_D3 decreases PTH production directly by decreasing PTH transcription [53], and indirectly by increasing serum calcium levels. Similarly, we found that LP intake increased *Cyp27b1* renal expression (**S3B Fig**), leading to increased serum calcium levels and subsequent suppression of serum PTH (**S3C and S3D Fig**). Together, these findings are consistent with previous studies [5,22,50–53] showing that restriction of dietary phosphorus in normal mice induces a decrease in the serum concentrations of Pi and FGF23, and stimulates renal 1,25(OH)_2_D production by increasing *1α-hydroxylase* mRNA expression.

To examine the effect of phosphorus deficiency on red blood cell production, we first determined the hematological profile of mice fed LP diet compared to mice fed normal phosphorus diet. Our data show that mice fed a LP diet for 2 weeks had increased RBC counts (**Fig 7C**), hemoglobin concentration (**Fig 7D**), and hematocrit (**Fig 7E**), compared to mice fed normal diet. We next analyzed the erythroid progenitors in the bone marrow using flow cytometry and double labelling with CD71 and TER119. Mice fed LP diet had increased number of erythroid progenitors, particularly pro-erythroblasts (Pro-E) and polychromatic erythroblasts (**S1 Table**) compared to the normal group. However, the number of erythroid-burst forming units (BFU-E) in the bone marrow, which precede pro-erythroblasts, was reduced in the low phosphorus-fed group (**Fig 7F**), suggesting that the reduced counts of BFU-E colonies may be due to accelerated differentiation to pro-erythroblast stage in the LP group. Renal expression and circulating levels of EPO were decreased in mice fed LP diet (**Fig 7G and 7H**), most likely due to a feedback mechanism that signals the kidneys to turn off Epo secretion when red blood cell production is sufficient. Therefore, our data suggest that the observed erythrocytosis in mice fed LP diet is caused by alteration of a mechanism intrinsic to erythroid progenitors. Erythrocytosis in the LP diet mice may be mediated in part by a decrease in the RBC destruction rate. However, mRNA expression of heme oxygenase (*Hmox*), an essential enzyme for heme catabolism and iron recycling, was significantly increased in spleen of mice fed LP diet (**Fig 7I**). High levels of *Hmox-1* are detected in tissues that degrade aged red blood cells such as the spleen and liver. Thus, the increase in splenic *Hmox* is indicative of increased erythrocyte clearance in non-inflammatory conditions.

**Fig 8:**
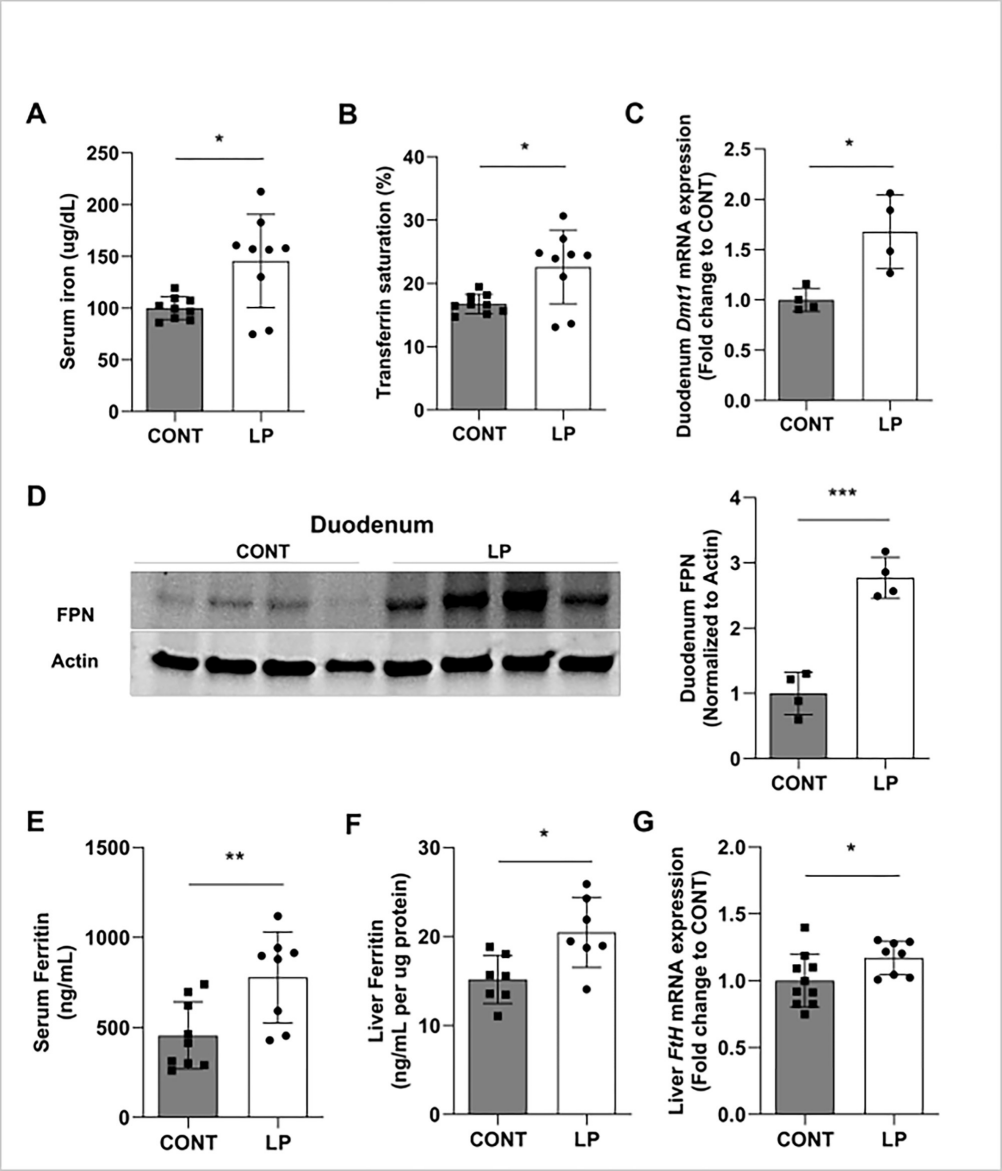
Effect of dietary phosphorus deficiency on iron homeostasis and inflammation. Eight-week old C57BL/6J male mice were fed a diet containing 0.02% inorganic phosphorus (LP) for 2 weeks and compared to C57BL/6J male mice fed normal phosphorus diet (0.6% Pi; CONT). Serum and tissue samples were collected at the end of the experiment. **(A-B)** Serum levels of **(A)** iron and **(B)** transferrin saturation. **(C)** Quantitative real-time RT-PCR for duodenal expression of *Dmt1*. Data are expressed as fold change (2^-ΔΔCt^) relative to housekeeping gene *Hprt*. **(D)** Protein expression of Ferroportin (FPN) in total duodenum, shown in representative image of western blot and quantitative graph. Abundance of FPN protein was normalized with Actin. **(E)** Serum concentration of Ferritin. Samples were measured in duplicates. **(F)** Liver Ferritin protein measured by ELISA in liver lysates. **(G)** Quantitative real-time RT-PCR for liver expression of *Ferritin H*. Data are expressed as fold change (2^-ΔΔCt^) relative to housekeeping gene *Hprt*. Data are represented as mean ± SD. (n = 7–10 per group for A, B, E, F, G; n = 4 per group for C and D). All data were analyzed for normality with Shapiro-Wilk test and homogeneity of variance by F test. For samples with normal distribution, unpaired t test was performed compared to CONT (C, D, E, F, G). When the samples were in normal distribution but not in homogeneity of variance, the data were analyzed by Welch’s t test (A, B). *P < 0.05 and ***P < 0.001 compared to CONT (control diet).

We also investigated the effect of phosphate restriction on iron metabolism. After 2 weeks of LP diet, serum iron and transferrin saturation levels were significantly increased (**Fig 8A and 8B**). Hepcidin inhibits iron absorption in enterocytes by downregulating divalent metal transporter 1 (DMT1), a protein that facilitates iron uptake, and suppressing ferroportin (FPN), [54,55]. In the liver, hepcidin controls iron efflux and storage [27,56]. Consistent with this, we found that the rise in serum iron levels in the LP group was associated with upregulation of DMT1 mRNA and FPN protein in the duodenum (**Figs 8C, 8D** and **S2A**), that are indicative of increased dietary iron absorption. Functional iron uptake studies are needed to confirm changes in iron absorption. Moreover, our data show increased serum ferritin (**Fig 8E**), liver ferritin protein (**Fig 8F**), and liver ferritin H mRNA expression (**Fig 8G**) in mice fed LP diet, which correspond to increased serum iron levels. Liver iron deposition was decreased in mice fed LP diet (**S4A and S4B Fig**). However, phosphorus restriction had no effect on hepatic expression of hepcidin (**S4C Fig**) or on pro-inflammatory cytokines such as IL-6, TNF-α, and IL-1β (**S4D–S4F Fig**).

### Red blood cell production and iron regulation in a mouse model of XLH

**Fig 9:**
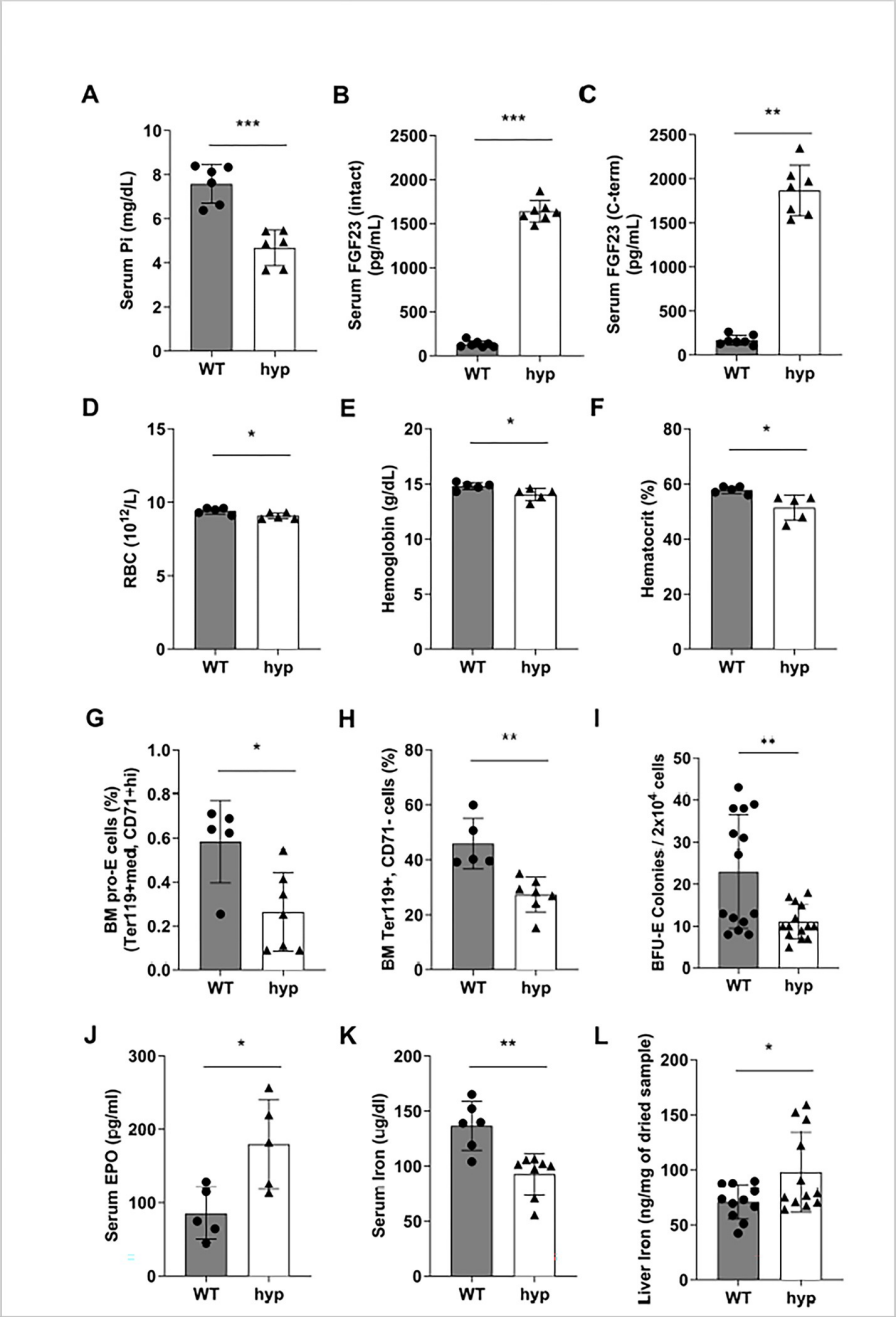
Red blood cell production and iron homeostasis in hyp mice. Analysis of hematological and iron parameters in eight-week-old male mice carrying a mutation in the *Phex* gene (hyp mice) compared to age and sex matched wild-type littermates (control). **(A-C)** Serum concentration of **(A)** Phosphorus**, (B)** intact FGF23, and **(C)** C-terminal FGF23. **(D-F)** Circulating red blood cell parameters. **(D)** Red Blood Cells (RBCs), **(E)** Hemoglobin (Hgb), **(F)** Hematocrit (Hct). **(G-H)** Flow cytometry analysis of bone marrow erythroid progenitor cells from hyp and wild-type (control) mice. Percent of **(G)** pro-erythroblasts (pro-E) stained positive for Ter119^med^ and CD71^high^ and **(H)** terminally differentiated erythroid cells stained positive for Ter119^high^ and negative for CD71. **(I)** Colony forming unit assay. Bone marrow cells were isolated from femora and tibiae, cultured for 12 days on methylcellulose medium, and counted for erythroid progenitor cells, burst forming unit-erythroid (BFU-E). **(J-L)** Serum concentration of **(J)** EPO and **(K)** iron. **(L)** Liver iron content was measured by the ferrozine colorimetric assay and normalized to weight (mg) of dried tissue sample. Tissues were weighed at collection and their weight was normalized to total body weight of the animal and represented as percentage. Samples were measured in duplicates. Data are represented as mean ± SD. (n = 5–8 per group). All data were analyzed for normality with Shapiro-Wilk test and homogeneity of variance by F test. For samples with normal distribution, unpaired t test was performed compared to WT (B, C, D, E, G, H, I, J, K). When the samples were in normal distribution but not in homogeneity of variance, the data were analyzed by Welch’s t test (F, L). When samples were not normally distributed for parametric analysis, the data were analyzed by a non-parametric Mann-Whitney test (A). *P < 0.05, **P < 0.01, ***P < 0.001 compared to wild-type (WT).

Because dietary phosphorus intake affects production and circulating levels of FGF23, in this study we also investigated if red blood cell production and iron homeostasis are regulated directly by phosphorus or through FGF23. The hyp mouse is the murine model of X-linked hypophosphatemia (XLH). XLH is an inherited disease of phosphate metabolism in which inactivating mutations of the Phosphate-Regulating Endopeptidase Homolog, X-Linked (*PHEX*) gene lead to local and systemic effects including hypophosphatemia, bone defects such as rickets and osteomalacia, and elevated levels of FGF23. Our data confirmed the hypophosphatemia and high FGF23 levels in hyp mice (**Fig 9A–9C**). The percentage of intact to C-terminal FGF23 is shown in **S1C Fig**. Importantly, we found that hyp mice exhibit anemia, as determined by low circulating red blood cells (**Fig 9D**), hemoglobin (**Fig 9E**), and hematocrit (**Fig 9F**) compared to wild-type littermate mice. Moreover, hyp mice had decreased number of erythroid progenitors, both early (pro-erythroblasts) (**Fig 9G**) and terminally differentiated erythroblasts (**Fig 9H**) in bone marrow and formed significantly less BFU-E colonies (**Fig 9I**) compared to control mice. This reduction in circulating red blood cells and bone marrow progenitors resulted in increased secretion of EPO (**Fig 9J**) in order to increase production of red blood cells and meet the body’s demands. Serum iron levels (**Fig 9K**) were also decreased in hyp mice in comparison to wild-type mice and this drop in circulating iron was associated with increased liver iron retention (**Fig 9L**).

## Discussion

Our study is the first to demonstrate that elevated FGF23 negatively affects red blood cell production and iron homeostasis independent of phosphorus dietary intake and circulating levels. We previously reported that high FGF23 levels contribute to the development of anemia, iron deficiency, and inflammation, and that inhibition of FGF23 signaling or genetic deletion of *Fgf23* results in increased erythropoiesis and serum iron levels, as well as amelioration of inflammation in mice with renal failure or normal kidney function [6,7,57]. The growing consumption of a phosphorus-rich diet in the form of processed foods and phosphorus-containing food additives, has increased the prevalence of hyperphosphatemia [58]. Elevated serum phosphate levels are associated with adverse outcomes, including CKD and progression to renal failure, cardiovascular disease, and mortality, even in individuals with normal kidney function [59–63]. These conditions are associated with several comorbidities such as anemia and inflammation. The contribution of phosphorus to red blood cell production and iron regulation has been addressed only in a small number of studies, mainly with regards to high phosphorus levels and their association with a greater likelihood for development of anemia [10,12,48]. However, the role of increased FGF23 levels by high phosphorus in these individuals was not taken into consideration in these studies. An even smaller cohort of studies, investigated the effect of dietary phosphorus deficiency on erythropoiesis with variable results, with one study reporting that a low phosphorus (LP) diet results in low serum phosphorus levels, decreased red blood cells and hemoglobin, and microcytosis [11], whereas another study reported no effect on the number of RBCs [64]. Both of these studies were performed in cows and neither of them measured FGF23 levels. Since higher phosphorus induces elevation in FGF23, it is challenging to distinguish if the effect on erythropoiesis and iron metabolism is due to high phosphorus or high FGF23. Therefore, it remains unclear if phosphorus affects erythropoiesis directly or indirectly through the actions of other factors, such as FGF23, and whether the actions of FGF23 on erythropoiesis and iron regulation are dependent or independent of phosphorus levels. To address this, in the present study we investigated the effect of a) phosphorus overload and b) phosphorus restriction on erythropoiesis and iron homeostasis in association with FGF23.

Our data show that intake of a phosphate-rich diet results in inflammation-induced hypoferremia and ineffective erythropoiesis leading to anemia through the actions of elevated FGF23 on erythroid progenitors, in the absence of hyperphosphatemia and despite increased EPO secretion. In our study, serum phosphate levels remained stable after 2 weeks of high dietary phosphorus intake. We expect that serum phosphate levels did rise short term by the increased phosphorus load, as it is reported in previous studies in which high phosphorus diet was given for 3 or 5 days [5,50]. However, during 2 weeks of increased phosphorus intake in our study, elevated FGF23 combated hyperphosphatemia by inducing effective excretion of phosphate excess.

Phosphate is necessary in red blood cells for production of 2,3-diphosphoglycerate (2,3-DPG), which facilitates release of oxygen from hemoglobin for delivery to the tissues of the body by decreasing the oxygen-binding capacity of hemoglobin. [65,66]. 2,3-DPG increases in response to either hypoxia.[67] or EPO treatment [68–70]. Low serum phosphate levels reduce 2,3-DPG concentration in RBCs [46], and they are associated with acute hemolytic anemia [44,45], whereas parenteral Pi administration can restore 2,3-DPG levels to normal and correct hemolytic anemia [44,45]. Similarly, plasma 2,3-DPG concentration is increased in rats fed a high phosphorus diet [71]. Although in our study we did not measure 2,3-DPG, the increase in circulating and renal EPO in mice fed high phosphorus diet suggests that oxygen delivery was not increased and, thus, 2,3-DPG must not have been affected by high phosphorus intake.

It has also been reported that phosphate deficiency may decrease the life span of red blood cells by negatively affecting the rate of erythrocyte glycolysis, which is critical for cellular integrity [8,46,47], whereas *in vitro* studies showed that increased concentration of inorganic Pi stimulates glucose utilization of human erythrocytes [9]. It is not yet determined whether increased dietary phosphorus increases the life span of red blood cells *in vivo*. We found that mice fed high phosphorus diet had low RBCs, hemoglobin, and hematocrit, and increased expression of *Hmox-1*. Hmox-1 is critical for heme catabolism and iron recycling, and high levels of *Hmox-1* are detected in tissues that degrade aged red blood cells such as the spleen and liver [49]. Collectively, our data showing increased *Hmox-1* and tissue iron deposition, and decreased RBCs despite increased EPO secretion, suggest that phosphorus overload results in increased destruction of RBCs leading to increased tissue iron overload from the lysed erythrocytes. Moreover, it has been shown that iron accumulation in the kidney during RBC destruction represents a protective mechanism for hepatocytes from iron overload [72]. Taken together, our data does not support increase in RBC life span by high phosphorus supplementation.

In our study we also found that *Erfe* expression was upregulated in the bone marrow of mice fed 1.65% Pi diet compared to the control diet group. In contrast to Erfe’s role as a hepcidin suppressor, hepcidin was not suppressed in our high phosphorus fed group. One plausible explanation is that overproduction of pro-inflammatory cytokines and the presence of persistent inflammation have blunted the effect of Erfe on hepcidin. This is consistent with the presence of anemia of inflammation especially in the acute form [73,74]. Moreover, since *Erfe* is expressed in erythroblasts, it is possible that when erythroblast numbers are low, as it is the case in our study, *Erfe* expression is not sufficient to suppress hepcidin. A recent study has shown that *Erfe* is also expressed in osteoblasts [75], suggesting that bone cells play a role in the regulation of *Erfe*, and, possibly, have an effect on hepcidin secretion. The latter warrants further investigation.

Consistent with published data reporting that phosphorus overload induces inflammation [24], we show that phosphorus supplementation stimulates hepcidin secretion and results in inflammation-induced hypoferremia. Hyperphosphatemia has been associated with increased incidence of infection in dialysis patients [76], possibly due to increased FGF23 levels. It has been shown that elevated FGF23 in mice with CKD impairs neutrophil recruitment and disrupts the host defense mechanism in infection, whereas neutralization of FGF23 restores neutrophil recruitment and protects the host against pathogens during infection [77]. In our study, we found that increased phosphorus intake results in decreased neutrophil numbers (Fig 5H), most likely due to elevated FGF23. The exact mechanism of how FGF23 may regulate iron homeostasis is still under investigation. FGF23 may regulate iron metabolism indirectly through erythropoiesis, although a direct mechanism is also possible. In our previous work [6,57], we showed that FGF23 is a negative regulator of erythropoiesis and contributes to the red cell apoptosis. This may decrease the overall level of red cells and, therefore, decrease the pool of iron that can be recycled to the blood after erythrophagocytosis. Moreover, high FGF23 levels stimulate hepatic inflammation which, in turn, can stimulate iron deficiency that can feed back to the bone to stimulate FGF23 production. It is also possible that high phosphate diet-induced inflammation may mediate the adverse effects on iron status and erythropoiesis. However, we have previously shown that global *Fgf23* knockout mice that have hyperphosphatemia and inflammation, exhibit increased erythropoiesis and hematopoietic stem cells not only postnatally but also prenatally [57], indicating that FGF23 can act directly on the erythroid progenitors.

Another mechanism connecting iron and high phosphate in infection is through increased circulating non-transferrin-bound iron (NTBI). During infection, increased secretion of hepcidin contributes to the host defense action [78], not only by sequestering iron in macrophages to limit iron availability to the invading microbes that is needed for their growth, but also by eliminating NTBI, which can be more readily utilized by pathogens than transferrin-bound iron [79]. Circulating NTBI usually increases when the binding capacity of transferrin is exceeded (iron overload). In high phosphorus conditions, the iron-binding capacity of transferrin is decreased as iron binds to phosphorus instead of transferrin, consequently increasing the presence of available circulating NTBI (free iron) and favoring the growth of pathogens [80]. However, in our study we did not assess whether circulating NTBI is increased.

Our study has some limitations. First, it does not address a possible involvement of other secreted factors such as phosphatonins which may also be induced by the high phosphorus diet and may contribute to the observed changes in erythropoiesis and iron. A recent study has also suggested that indoxyl sulfate (IS), a uremic toxin that accumulates during CKD progression, affects cell viability and erythroid cell differentiation by impairing the differentiation of the erythroid progenitors (BFU-E) into RBCs and triggering apoptosis [81]. Moreover, it has been shown that 1,25(OH)2D induces erythroid cell proliferation, upregulates EPOR, and decreases hepcidin and inflammation [82,83]. Thus, it is possible that the effects we observe on erythropoiesis and iron status in our models may be, at least partially, mediated by changes in 1,25(OH)2D levels. Second, we only looked at one time point (2 weeks after high or low phosphorus diet, or hyp mice at 8 weeks of age) which makes it challenging to distinguish between primary effects of phosphorus intake and secondary effects due to feedback regulation.

However, a strength of our study is the comparison of the two mouse models both of which exhibit hypophosphatemia–either acquired induced by diet, or genetically-induced—but opposite levels of FGF23. The comparison of these 2 mouse models provided us with the unique opportunity to delineate whether phosphorus plays a role in the actions of FGF23 in red blood cell production and iron homeostasis or it has a role of its own.

Overall, the data presented here are consistent with our previously published studies showing that FGF23 is a negative regulator of erythropoiesis and that inhibition of Fgf23, genetically or pharmacologically, stimulates erythropoiesis [6,7,57]. Here we have shown that in the presence of normophosphatemia, dietary phosphorus overload through the actions of FGF23, results in off-target effect of inflammation-induced hypoferremia associated with tissue iron overload, and anemia caused by decreased production and increased destruction of red blood cells that cannot be corrected by increased EPO secretion. Therefore, even when phosphorus load does not result in increased serum phosphorus or mild elevation of serum phosphorus occurs within the normal range, phosphorus load stimulates FGF23 secretion resulting in several adverse outcomes, including anemia and iron deficiency. Excess dietary phosphorus intake has deleterious health effects such as disturbing regulation of phosphorus and mineral metabolism and contributing to vascular calcifications, bone loss, and renal failure. Increased consumption of phosphorus-containing processed food is, therefore, a critical issue that needs to be addressed. Importantly, our findings clearly demonstrate that FGF23 acts independent of phosphorus levels to regulate erythropoiesis. To our knowledge, this is the first study that examines erythropoiesis in a mouse model of XLH and provides evidence of a phosphorus-independent role of FGF23 in erythropoiesis and iron metabolism.

## Supporting information

S1 Table Flow cytometry analysis of bone marrow erythroid cells. Bone marrow cell suspensions were prepared from dissected tibiae and femora from mice fed control (0.6% Pi) or low phosphorus diet (0.02% Pi), as described in Coe *et al* 2014. For immunostaining, cells were first incubated with CD16/32 antibody to block mouse Fc receptor and reduce non-specific binding. Erythroid lineage was assessed using Ter119-APC / CD71-PE markers combined with the forward scatter (FSC) properties, as described in Asari S *et al* 2005 Exp Hematol, and Koulnis M *et al* 2011 J Vis Exp. Labeled cells were then analyzed by flow cytometry. Appropriate isotype controls were kept for each set. Forward and side scatter patterns were gated excluding the debris. A total of 20,000 events were collected and analyzed using FlowJo software.
(PDF)

S1 Fig Relative percentage of intact FGF23 to C-terminal FGF23. Relative percentage of intact FGF23 relative to C-terminal FGF23 in serum. (A) C57BL/6J male mice were fed a diet containing 0.6% inorganic phosphorus (Pi), 1.2% Pi, or 1.65% Pi for 2 weeks. (B) Eight-week-old male mice were fed a diet containing 0.02% or 0.6% inorganic phosphorus (LP) for 2 weeks. (C) Eight-week-old male mice carrying a mutation in the Phex gene (hyp mice) compared to age and sex matched wild-type littermates (control). Data are represented as mean ± SD. All data were analyzed for normality with Shapiro-Wilk test, homogeneity of variance by F test. Samples were in normal distribution and equal variance. One-way ANOVA with Dunnett’s multiple comparison test was performed (A), or unpaired t test was performed compared to WT (B, C). *P <0.05, ****P <0.0001 compared to CONT (control diet).
(PDF)

S2 Fig Protein expression of ferroportin in duodenum and spleen in mice fed high (1.2% or 1.65%) or low phosphate (0.02%) diets compared to control diet (0.6% Pi). Original uncropped Western Blot images of protein expression of Ferroportin (FPN) and actin in **(A)** duodenum and **(B)** spleen.
(PDF)

S3 Fig Acquired phosphorus deficiency by low dietary phosphorus intake. Eight-week old C57BL/6J male mice were fed a diet containing 0.02% inorganic phosphorus (LP) for 2 weeks and compared to age matched C57BL/6J male mice fed normal phosphorus diet (0.6% Pi; CONT). Serum and tissue samples were collected at the end of the experiment. **(A)** Serum phosphorus levels, **(B)** Quantitative real-time RT-PCR for renal *Cyp27b1* expression. Data are expressed as fold change (2-ΔΔCt) relative to housekeeping gene *Hprt*. **(C)** Serum calcium levels, and **(D)** Circulating PTH levels measured by ELISA. All data were analyzed for normality with Shapiro-Wilk test, homogeneity of variance by F test, and unpaired t test was performed. For samples with normal distribution and equal variances, unpaired t test was performed compared to WT (A). When the samples were in normal distribution but not in homogeneity of variance, the data were analyzed by Welch’s t test (B, C, D). *P < 0.05, **P < 0.01, ***P <0.001 compared to CONT (control diet).
(PDF)

S4 Fig Effects of dietary phosphorus restriction on hepatic iron content and inflammatory markers. Eight-week old C57BL/6J male mice were fed a diet containing 0.02% inorganic phosphorus (LP) for 2 weeks and compared to age matched C57BL/6J male mice fed normal phosphorus diet (0.6% Pi; CONT). Liver samples were collected at the end of the experiment. (A) Iron content in the liver assessed by the ferrozine colorimetric assay, (B) Representative images of Prussian blue staining for liver iron accumulation (Blue, iron; pink, hepatic nuclei and cytoplasm. (C-F) Quantitative real-time RT-PCR for hepatic inflammatory marker expression. Data are expressed as fold change (2-ΔΔCt) relative to housekeeping gene Hprt. (C) Hepcidin, (D) IL-6, (E) TNF-α, and (F) IL-1β. For samples with normal distribution and equal variances, unpaired t test was performed compared to WT (A, C, D, E). When the samples were in normal distribution but not in homogeneity of variance, the data were analyzed by Welch’s t test (F). *P <0.05 compared to CONT (control diet).
(PDF)

## References

[1] Berndt TJ, Schiavi S, and Kumar R. "Phosphatonins" and the regulation of phosphorus homeostasis. *Am J Physiol Renal Physiol*. 2005;289(6):F1170–82. doi: 10.1152/ajprenal.00072.2005 16275744

[2] Shaikh A, Berndt T, and Kumar R. Regulation of phosphate homeostasis by the phosphatonins and other novel mediators. *Pediatr Nephrol*. 2008;23(8):1203–10. doi: 10.1007/s00467-008-0751-z 18288501 PMC2441591

[3] Takeda E, Yamamoto H, Nashiki K, Sato T, Arai H, and Taketani Y. Inorganic phosphate homeostasis and the role of dietary phosphorus. *J Cell Mol Med*. 2004;8(2):191–200. doi: 10.1111/j.1582-4934.2004.tb00274.x 15256067 PMC6740209

[4] Kawamura H, Tanaka S, Ota Y, Endo S, Tani M, Ishitani M, et al. Dietary intake of inorganic phosphorus has a stronger influence on vascular-endothelium function than organic phosphorus. *J Clin Biochem Nutr*. 2018;62(2):167–73. doi: 10.3164/jcbn.17-97 29610557 PMC5874240

[5] Perwad F, Azam N, Zhang MY, Yamashita T, Tenenhouse HS, and Portale AA. Dietary and serum phosphorus regulate fibroblast growth factor 23 expression and 1,25-dihydroxyvitamin D metabolism in mice. *Endocrinology*. 2005;146(12):5358–64. doi: 10.1210/en.2005-0777 16123154

[6] Agoro R, Montagna A, Goetz R, Aligbe O, Singh G, Coe LM, et al. Inhibition of fibroblast growth factor 23 (FGF23) signaling rescues renal anemia. *FASEB J*. 2018;32(7):3752–64. doi: 10.1096/fj.201700667R 29481308 PMC5998980

[7] Agoro R, Park MY, Le Henaff C, Jankauskas S, Gaias A, Chen G, et al. C-FGF23 peptide alleviates hypoferremia during acute inflammation. *Haematologica*. 2021;106(2):391–403. doi: 10.3324/haematol.2019.237040 32193252 PMC7849576

[8] Rose IA, and Warms JV. Control of glycolysis in the human red blood cell. *J Biol Chem*. 1966;241(21):4848–54. 4288723

[9] Rose IA, Warms JV, and O’Connell EL. Role of inorganic phosphate in stimulating the glucose utilization of human red blood cells. *Biochem Biophys Res Commun*. 1964;15(1):33–7. doi: 10.1016/0006-291x(64)90098-1 4220803

[10] Tran L, Batech M, Rhee CM, Streja E, Kalantar-Zadeh K, Jacobsen SJ, et al. Serum phosphorus and association with anemia among a large diverse population with and without chronic kidney disease. *Nephrol Dial Transplant*. 2016;31(4):636–45. doi: 10.1093/ndt/gfv297 26254460 PMC4805130

[11] Zhang Z, Bi M, Yang J, Yao H, Liu Z, and Xu S. Effect of phosphorus deficiency on erythrocytic morphology and function in cows. *J Vet Sci*. 2017;18(3):333–40. doi: 10.4142/jvs.2017.18.3.333 27586463 PMC5639086

[12] Kovesdy CP, Mucsi I, Czira ME, Rudas A, Ujszaszi A, Rosivall L, et al. Association of serum phosphorus level with anemia in kidney transplant recipients. *Transplantation*. 2011;91(8):875–82. doi: 10.1097/TP.0b013e3182111edf 21358369

[13] Agoro R, Taleb M, Quesniaux VFJ, and Mura C. Cell iron status influences macrophage polarization. *PLoS One*. 2018;13(5):e0196921. doi: 10.1371/journal.pone.0196921 29771935 PMC5957380

[14] Barry M, and Sherlock S. Measurement of liver-iron concentration in needle-biopsy specimens. *Lancet*. 1971;1(7690):100–3. doi: 10.1016/s0140-6736(71)90838-5 4099600

[15] Asari S, Sakamoto A, Okada S, Ohkubo Y, Arima M, Hatano M, et al. Abnormal erythroid differentiation in neonatal bcl-6-deficient mice. *Exp Hematol*. 2005;33(1):26–34. doi: 10.1016/j.exphem.2004.10.001 15661395

[16] Koulnis M, Pop R, Porpiglia E, Shearstone JR, Hidalgo D, and Socolovsky M. Identification and analysis of mouse erythroid progenitors using the CD71/TER119 flow-cytometric assay. *J Vis Exp*. 2011(54). doi: 10.3791/2809 21847081 PMC3211121

[17] Gattineni J, Bates C, Twombley K, Dwarakanath V, Robinson ML, Goetz R, et al. FGF23 decreases renal NaPi-2a and NaPi-2c expression and induces hypophosphatemia in vivo predominantly via FGF receptor 1. *Am J Physiol Renal Physiol*. 2009;297(2):F282–91. doi: 10.1152/ajprenal.90742.2008 19515808 PMC2724258

[18] Hu MC, Shi M, and Moe OW. Role of alphaKlotho and FGF23 in regulation of type II Na-dependent phosphate co-transporters. *Pflugers Arch*. 2019;471(1):99–108. 30506274 10.1007/s00424-018-2238-5 PMC6324980

[19] Estepa JC, Aguilera-Tejero E, Lopez I, Almaden Y, Rodriguez M, and Felsenfeld AJ. Effect of phosphate on parathyroid hormone secretion in vivo. *J Bone Miner Res*. 1999;14(11):1848–54. doi: 10.1359/jbmr.1999.14.11.1848 10571684

[20] Martin DR, Ritter CS, Slatopolsky E, and Brown AJ. Acute regulation of parathyroid hormone by dietary phosphate. *Am J Physiol Endocrinol Metab*. 2005;289(4):E729–34. doi: 10.1152/ajpendo.00065.2005 15914507

[21] Murer H, Hernando N, Forster I, and Biber J. Proximal tubular phosphate reabsorption: molecular mechanisms. *Physiol Rev*. 2000;80(4):1373–409. doi: 10.1152/physrev.2000.80.4.1373 11015617

[22] Portale AA, Halloran BP, and Morris RC Jr. Physiologic regulation of the serum concentration of 1,25-dihydroxyvitamin D by phosphorus in normal men. *J Clin Invest*. 1989;83(5):1494–9. doi: 10.1172/JCI114043 2708521 PMC303852

[23] Portale AA, Halloran BP, Murphy MM, and Morris RC Jr. Oral intake of phosphorus can determine the serum concentration of 1,25-dihydroxyvitamin D by determining its production rate in humans. *J Clin Invest*. 1986;77(1):7–12. doi: 10.1172/JCI112304 3753709 PMC423300

[24] Yamada S, Tokumoto M, Tatsumoto N, Taniguchi M, Noguchi H, Nakano T, et al. Phosphate overload directly induces systemic inflammation and malnutrition as well as vascular calcification in uremia. *Am J Physiol Renal Physiol*. 2014;306(12):F1418–28. doi: 10.1152/ajprenal.00633.2013 24808541

[25] Nemeth E, Valore EV, Territo M, Schiller G, Lichtenstein A, and Ganz T. Hepcidin, a putative mediator of anemia of inflammation, is a type II acute-phase protein. *Blood*. 2003;101(7):2461–3. doi: 10.1182/blood-2002-10-3235 12433676

[26] Ganz T. Hepcidin, a key regulator of iron metabolism and mediator of anemia of inflammation. *Blood*. 2003;102(3):783–8. doi: 10.1182/blood-2003-03-0672 12663437

[27] Nemeth E, Tuttle MS, Powelson J, Vaughn MB, Donovan A, Ward DM, et al. Hepcidin regulates cellular iron efflux by binding to ferroportin and inducing its internalization. *Science*. 2004;306(5704):2090–3. doi: 10.1126/science.1104742 15514116

[28] Ganz T, and Nemeth E. Iron homeostasis in host defence and inflammation. *Nat Rev Immunol*. 2015;15(8):500–10. doi: 10.1038/nri3863 26160612 PMC4801113

[29] Farrow EG, Yu X, Summers LJ, Davis SI, Fleet JC, Allen MR, et al. Iron deficiency drives an autosomal dominant hypophosphatemic rickets (ADHR) phenotype in fibroblast growth factor-23 (Fgf23) knock-in mice. *Proc Natl Acad Sci U S A*. 2011;108(46):E1146–55. doi: 10.1073/pnas.1110905108 22006328 PMC3219119

[30] Collins JF, Franck CA, Kowdley KV, and Ghishan FK. Identification of differentially expressed genes in response to dietary iron deprivation in rat duodenum. *Am J Physiol Gastrointest Liver Physiol*. 2005;288(5):G964–71. doi: 10.1152/ajpgi.00489.2004 15637178

[31] Clinkenbeard EL, Farrow EG, Summers LJ, Cass TA, Roberts JL, Bayt CA, et al. Neonatal iron deficiency causes abnormal phosphate metabolism by elevating FGF23 in normal and ADHR mice. *J Bone Miner Res*. 2014;29(2):361–9. doi: 10.1002/jbmr.2049 23873717 PMC5240191

[32] Wolf M, Chertow GM, Macdougall IC, Kaper R, Krop J, and Strauss W. Randomized trial of intravenous iron-induced hypophosphatemia. *JCI Insight*. 2018;3(23). doi: 10.1172/jci.insight.124486 30518682 PMC6328019

[33] Wolf M, Koch TA, and Bregman DB. Effects of iron deficiency anemia and its treatment on fibroblast growth factor 23 and phosphate homeostasis in women. *J Bone Miner Res*. 2013;28(8):1793–803. doi: 10.1002/jbmr.1923 23505057

[34] Block GA, Fishbane S, Rodriguez M, Smits G, Shemesh S, Pergola PE, et al. A 12-week, double-blind, placebo-controlled trial of ferric citrate for the treatment of iron deficiency anemia and reduction of serum phosphate in patients with CKD Stages 3–5. *Am J Kidney Dis*. 2015;65(5):728–36. doi: 10.1053/j.ajkd.2014.10.014 25468387

[35] Lee CT, Wu IW, Chiang SS, Peng YS, Shu KH, Wu MJ, et al. Effect of oral ferric citrate on serum phosphorus in hemodialysis patients: multicenter, randomized, double-blind, placebo-controlled study. *J Nephrol*. 2015;28(1):105–13. doi: 10.1007/s40620-014-0108-6 24840781

[36] Yokoyama K, Hirakata H, Akiba T, Sawada K, and Kumagai Y. Effect of oral JTT-751 (ferric citrate) on hyperphosphatemia in hemodialysis patients: results of a randomized, double-blind, placebo-controlled trial. *Am J Nephrol*. 2012;36(5):478–87. doi: 10.1159/000344008 23147696

[37] Weiss G. Iron metabolism in the anemia of chronic disease. *Biochim Biophys Acta*. 2009;1790(7):682–93. doi: 10.1016/j.bbagen.2008.08.006 18786614

[38] Nemeth E, and Ganz T. Anemia of inflammation. *Hematol Oncol Clin North Am*. 2014;28(4):671–81, vi. doi: 10.1016/j.hoc.2014.04.005 25064707 PMC4115203

[39] Macdougall IC, and Cooper AC. Erythropoietin resistance: the role of inflammation and pro-inflammatory cytokines. *Nephrol Dial Transplant*. 2002;17 Suppl 11:39–43. doi: 10.1093/ndt/17.suppl_11.39 12386257

[40] Means RT Jr, and Krantz SB. Progress in understanding the pathogenesis of the anemia of chronic disease. *Blood*. 1992;80(7):1639–47. 1391934

[41] Stoffel NU, Lazrak M, Bellitir S, Mir NE, Hamdouchi AE, Barkat A, et al. The opposing effects of acute inflammation and iron deficiency anemia on serum hepcidin and iron absorption in young women. *Haematologica*. 2019;104(6):1143–9. doi: 10.3324/haematol.2018.208645 30630976 PMC6545852

[42] Means RT, Jr., and Krantz SB. Inhibition of human erythroid colony-forming units by interferons alpha and beta: differing mechanisms despite shared receptor. *Exp Hematol*. 1996;24(2):204–8. 8641342

[43] Dai CH, Price JO, Brunner T, and Krantz SB. Fas ligand is present in human erythroid colony-forming cells and interacts with Fas induced by interferon gamma to produce erythroid cell apoptosis. *Blood*. 1998;91(4):1235–42. 9454753

[44] Jacob HS, and Amsden T. Acute hemolytic anemia with rigid red cells in hypophosphatemia. *N Engl J Med*. 1971;285(26):1446–50. doi: 10.1056/NEJM197112232852602 5122895

[45] Sheldon GF, and Grzyb S. Phosphate depletion and repletion: relation to parenteral nutrition and oxygen transport. *Ann Surg*. 1975;182(6):683–9. doi: 10.1097/00000658-197512000-00004 811182 PMC1343961

[46] Lichtman MA, and Miller DR. Erythrocyte glycolysis, 2,3-diphosphoglycerate and adenosine triphosphate concentration in uremic subjects: relationship to extracellular phosphate concentration. *J Lab Clin Med*. 1970;76(2):267–79. 5434006

[47] Lichtman MA, Miller DR, Cohen J, and Waterhouse C. Reduced red cell glycolysis, 2, 3-diphosphoglycerate and adenosine triphosphate concentration, and increased hemoglobin-oxygen affinity caused by hypophosphatemia. *Ann Intern Med*. 1971;74(4):562–8. doi: 10.7326/0003-4819-74-4-562 4994546

[48] Wojcicki JM. Hyperphosphatemia is associated with anemia in adults without chronic kidney disease: results from the National Health and Nutrition Examination Survey (NHANES): 2005–2010. *BMC Nephrol*. 2013;14:178. doi: 10.1186/1471-2369-14-178 23965134 PMC3765322

[49] Ryter SW, Alam J, and Choi AM. Heme oxygenase-1/carbon monoxide: from basic science to therapeutic applications. *Physiol Rev*. 2006;86(2):583–650. doi: 10.1152/physrev.00011.2005 16601269

[50] Vervloet MG, van Ittersum FJ, Buttler RM, Heijboer AC, Blankenstein MA, and ter Wee PM. Effects of dietary phosphate and calcium intake on fibroblast growth factor-23. *Clin J Am Soc Nephrol*. 2011;6(2):383–9. doi: 10.2215/CJN.04730510 21030580 PMC3052230

[51] Ferrari SL, Bonjour JP, and Rizzoli R. Fibroblast growth factor-23 relationship to dietary phosphate and renal phosphate handling in healthy young men. *J Clin Endocrinol Metab*. 2005;90(3):1519–24. doi: 10.1210/jc.2004-1039 15613425

[52] Tsai WC, Wu HY, Peng YS, Hsu SP, Chiu YL, Chen HY, et al. Effects of lower versus higher phosphate diets on fibroblast growth factor-23 levels in patients with chronic kidney disease: a systematic review and meta-analysis. *Nephrol Dial Transplant*. 2018;33(11):1977–83. doi: 10.1093/ndt/gfy005 29420827

[53] Silver J, Russell J, and Sherwood LM. Regulation by vitamin D metabolites of messenger ribonucleic acid for preproparathyroid hormone in isolated bovine parathyroid cells. *Proc Natl Acad Sci U S A*. 1985;82(12):4270–3. doi: 10.1073/pnas.82.12.4270 3858880 PMC397979

[54] Brasse-Lagnel C, Karim Z, Letteron P, Bekri S, Bado A, and Beaumont C. Intestinal DMT1 cotransporter is down-regulated by hepcidin via proteasome internalization and degradation. *Gastroenterology*. 2011;140(4):1261–71 e1. doi: 10.1053/j.gastro.2010.12.037 21199652

[55] Mena NP, Esparza A, Tapia V, Valdes P, and Nunez MT. Hepcidin inhibits apical iron uptake in intestinal cells. *Am J Physiol Gastrointest Liver Physiol*. 2008;294(1):G192–8. doi: 10.1152/ajpgi.00122.2007 17962361

[56] Ganz T, and Nemeth E. Hepcidin and iron homeostasis. *Biochim Biophys Acta*. 2012;1823(9):1434–43. doi: 10.1016/j.bbamcr.2012.01.014 22306005 PMC4048856

[57] Coe LM, Madathil SV, Casu C, Lanske B, Rivella S, and Sitara D. FGF-23 is a negative regulator of prenatal and postnatal erythropoiesis. *J Biol Chem*. 2014;289(14):9795–810. doi: 10.1074/jbc.M113.527150 24509850 PMC3975025

[58] Uribarri J, and Calvo MS. Dietary phosphorus excess: a risk factor in chronic bone, kidney, and cardiovascular disease? *Adv Nutr*. 2013;4(5):542–4. doi: 10.3945/an.113.004234 24038251 PMC3771143

[59] O’Seaghdha CM, Hwang SJ, Muntner P, Melamed ML, and Fox CS. Serum phosphorus predicts incident chronic kidney disease and end-stage renal disease. *Nephrol Dial Transplant*. 2011;26(9):2885–90. doi: 10.1093/ndt/gfq808 21292817 PMC3175050

[60] Aronson D, Kapeliovich M, Hammerman H, and Dragu R. The relation between serum phosphorus levels and clinical outcomes after acute myocardial infarction. *PLoS One*. 2013;8(3):e58348. doi: 10.1371/journal.pone.0058348 23505492 PMC3594318

[61] Dhingra R, Sullivan LM, Fox CS, Wang TJ, D’Agostino RB Sr, Gaziano JM, et al. Relations of serum phosphorus and calcium levels to the incidence of cardiovascular disease in the community. *Arch Intern Med*. 2007;167(9):879–85. doi: 10.1001/archinte.167.9.879 17502528

[62] Foley RN, Collins AJ, Herzog CA, Ishani A, and Kalra PA. Serum phosphorus levels associate with coronary atherosclerosis in young adults. *J Am Soc Nephrol*. 2009;20(2):397–404. doi: 10.1681/ASN.2008020141 18987306 PMC2637043

[63] Sim JJ, Bhandari SK, Smith N, Chung J, Liu IL, Jacobsen SJ, et al. Phosphorus and risk of renal failure in subjects with normal renal function. *Am J Med*. 2013;126(4):311–8. doi: 10.1016/j.amjmed.2012.08.018 23375678

[64] Grunberg W, Mol JA, and Teske E. Red blood cell phosphate concentration and osmotic resistance during dietary phosphate depletion in dairy cows. *J Vet Intern Med*. 2015;29(1):395–9. doi: 10.1111/jvim.12497 25407950 PMC4858069

[65] Benesch R, and Benesch RE. The effect of organic phosphates from the human erythrocyte on the allosteric properties of hemoglobin. *Biochem Biophys Res Commun*. 1967;26(2):162–7. doi: 10.1016/0006-291x(67)90228-8 6030262

[66] Mairbaurl H. Red blood cells in sports: effects of exercise and training on oxygen supply by red blood cells. *Front Physiol*. 2013;4:332. doi: 10.3389/fphys.2013.00332 24273518 PMC3824146

[67] Lenfant C, Torrance J, English E, Finch CA, Reynafarje C, Ramos J, et al. Effect of altitude on oxygen binding by hemoglobin and on organic phosphate levels. *J Clin Invest*. 1968;47(12):2652–6. doi: 10.1172/JCI105948 5725278 PMC297436

[68] Birgegard G, and Sandhagen B. Erythropoetin treatment can increase 2,3-diphosphoglycerate levels in red blood cells. *Scand J Clin Lab Invest*. 2001;61(5):337–40. doi: 10.1080/003655101316911369 11569479

[69] Crowley JP, Chazan JA, Metzger JB, Pono L, and Valeri CR. Blood rheology and 2,3-diphosphoglycerate levels after erythropoietin treatment. *Ann Clin Lab Sci*. 1993;23(1):24–32. 8430997

[70] Horina JH, Schwaberger G, Brussee H, Sauseng-Fellegger G, Holzer H, and Krejs GJ. Increased red cell 2,3-diphosphoglycerate levels in haemodialysis patients treated with erythropoietin. *Nephrol Dial Transplant*. 1993;8(11):1219–22. 8302459

[71] Nakao M, Yamamoto H, Nakahashi O, Ikeda S, Abe K, Masuda M, et al. Dietary phosphate supplementation delays the onset of iron deficiency anemia and affects iron status in rats. *Nutr Res*. 2015;35(11):1016–24. doi: 10.1016/j.nutres.2015.09.001 26475181

[72] Millot S, Delaby C, Moulouel B, Lefebvre T, Pilard N, Ducrot N, et al. Hemolytic anemia repressed hepcidin level without hepatocyte iron overload: lesson from Gunther disease model. *Haematologica*. 2017;102(2):260–70. 28143953 10.3324/haematol.2016.151621 PMC5286934

[73] Ganz T. Anemia of Inflammation. *N Engl J Med*. 2019;381(12):1148–57. doi: 10.1056/NEJMra1804281 31532961

[74] Weiss G, and Goodnough LT. Anemia of chronic disease. *N Engl J Med*. 2005;352(10):1011–23. doi: 10.1056/NEJMra041809 15758012

[75] Castro-Mollo M, Gera S, Ruiz-Martinez M, Feola M, Gumerova A, Planoutene M, et al. The hepcidin regulator erythroferrone is a new member of the erythropoiesis-iron-bone circuitry. *Elife*. 2021;10. doi: 10.7554/eLife.68217 34002695 PMC8205482

[76] Plantinga LC, Fink NE, Melamed ML, Briggs WA, Powe NR, and Jaar BG. Serum phosphate levels and risk of infection in incident dialysis patients. *Clin J Am Soc Nephrol*. 2008;3(5):1398–406. doi: 10.2215/CJN.00420108 18562596 PMC2518791

[77] Rossaint J, Oehmichen J, Van Aken H, Reuter S, Pavenstadt HJ, Meersch M, et al. FGF23 signaling impairs neutrophil recruitment and host defense during CKD. *J Clin Invest*. 2016;126(3):962–74. doi: 10.1172/JCI83470 26878171 PMC4767336

[78] Rodriguez R, Jung CL, Gabayan V, Deng JC, Ganz T, Nemeth E, et al. Hepcidin induction by pathogens and pathogen-derived molecules is strongly dependent on interleukin-6. *Infect Immun*. 2014;82(2):745–52. doi: 10.1128/IAI.00983-13 24478088 PMC3911381

[79] Stefanova D, Raychev A, Arezes J, Ruchala P, Gabayan V, Skurnik M, et al. Endogenous hepcidin and its agonist mediate resistance to selected infections by clearing non-transferrin-bound iron. *Blood*. 2017;130(3):245–57. doi: 10.1182/blood-2017-03-772715 28465342 PMC5520472

[80] Hilton RJ, Seare MC, Andros ND, Kenealey Z, Orozco CM, Webb M, et al. Phosphate inhibits in vitro Fe3+ loading into transferrin by forming a soluble Fe(III)-phosphate complex: a potential non-transferrin bound iron species. *J Inorg Biochem*. 2012;110:1–7. doi: 10.1016/j.jinorgbio.2012.02.017 22459167

[81] Hamza E, Vallejo-Mudarra M, Ouled-Haddou H, Garcia-Caballero C, Guerrero-Hue M, Santier L, et al. Indoxyl sulfate impairs erythropoiesis at BFU-E stage in chronic kidney disease. *Cell Signal*. 2023;104:110583. doi: 10.1016/j.cellsig.2022.110583 36596353

[82] Aucella F, Scalzulli RP, Gatta G, Vigilante M, Carella AM, and Stallone C. Calcitriol increases burst-forming unit-erythroid proliferation in chronic renal failure. A synergistic effect with r-HuEpo. *Nephron Clin Pract*. 2003;95(4):c121–7. doi: 10.1159/000074837 14694273

[83] Santoro D, Caccamo D, Lucisano S, Buemi M, Sebekova K, Teta D, et al. Interplay of vitamin D, erythropoiesis, and the renin-angiotensin system. *Biomed Res Int*. 2015;2015:145828. doi: 10.1155/2015/145828 26000281 PMC4427087

