# Supplementary material for: Phosphorus-independent role of FGF23 in erythropoiesis and iron homeostasis
Source: PLoS One. 2024 Dec 12;19(12):e0315228. doi: 10.1371/journal.pone.0315228 (PMC11637385; doi:10.1371/journal.pone.0315228)
Supplement: S1 Table — Bone marrow cell suspensions were prepared from dissected tibiae and femora from mice fed control (0.6% Pi) or low phosphorus diet (0.02% Pi), as described in Coe et al 2014. For immunostaining, cells were first incubated with CD16/32 antibody to block mouse Fc receptor and reduce non-specific binding. Erythroid lineage was assessed using Ter119-APC / CD71-PE markers combined with the forward scatter (FSC) properties, as described in Asari S et al 2005 Exp Hematol, and Koulnis M et al 2011 J Vis Exp. Labeled cells were then analyzed by flow cytometry. Appropriate isotype controls were kept for each set. Forward and side scatter patterns were gated excluding the debris. A total of 20,000 events were collected and analyzed using FlowJo software. (PDF) [file pone.0315228.s001.pdf]

Supplementary Table 1. Flow cytometry analysis of bone marrow erythroid cells.

Bone marrow cell suspensions were prepared from dissected tibiae and femora from mice fed control (0.6% Pi) or low phosphorus diet (0.02% Pi), as described in Coe et al 2014. For immunostaining, cells were first incubated with CD16/32 antibody to block mouse Fc receptor and reduce non-specific binding. Erythroid lineage was assessed using Ter119-APC / CD71-PE markers combined with the forward scatter (FSC) properties, as described in Asari S et al 2005 Exp Hematol, and Koulnis M et al 2011 J Vis Exp. Labeled cells were then analyzed by flow cytometry. Appropriate isotype controls were kept for each set. Forward and side scatter patterns were gated excluding the debris. A total of 20,000 events were collected and analyzed using FlowJo software.

	**CONT**	**LP**	**P value**
Pro-E cells (%)	0.267±0.069	0.470±0.126*	0.0299
Basophilic cells (%)	14.325±1.474	15.660±0.983	0.1952
Polychromatic cells (%)	5.710±0.907	7.902±0.854*	0.0249
Orthochromatic cells (%)	34.480±3.618	37.060±3.419	0.3302
