# Supplementary figures and images for: Phosphorus-independent role of FGF23 in erythropoiesis and iron homeostasis
Source: PLoS One. 2024 Dec 12;19(12):e0315228. doi: 10.1371/journal.pone.0315228 (PMC11637385; doi:10.1371/journal.pone.0315228)

# Supplementary Figure 1.

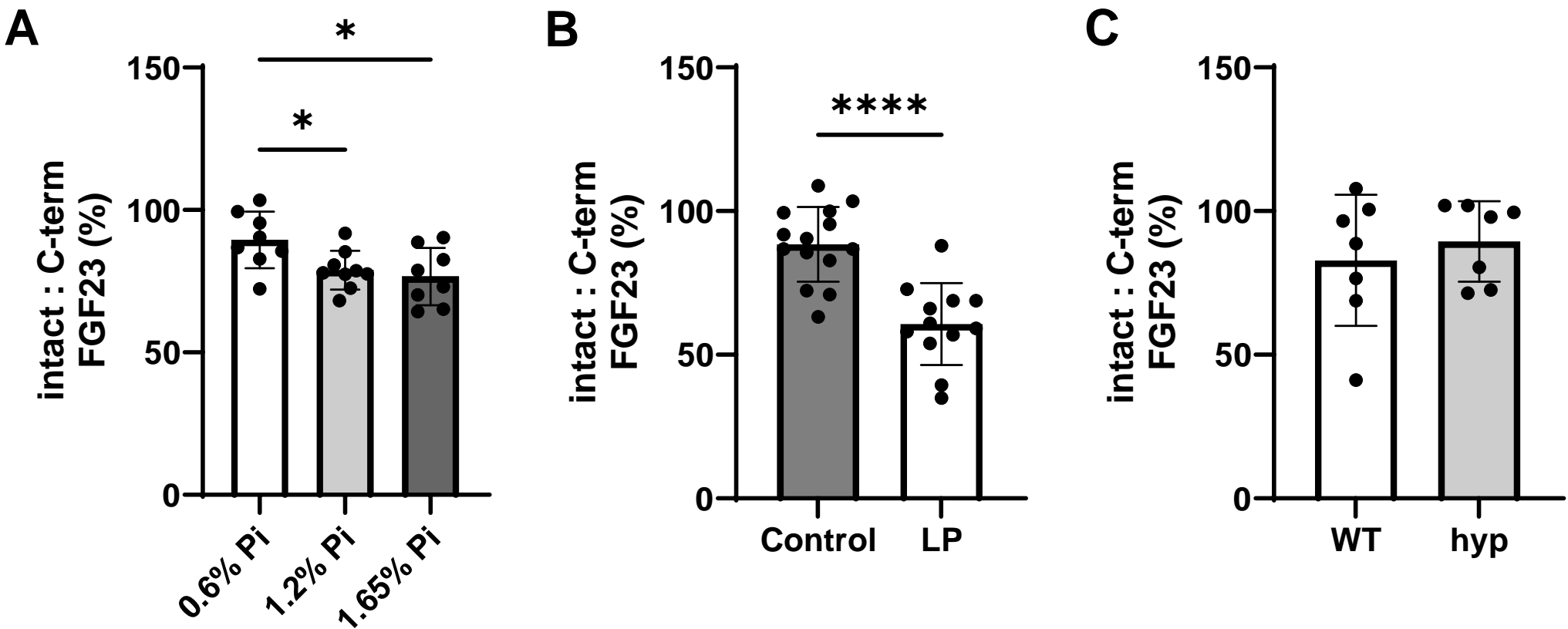

Supplement: S1 Fig — Relative percentage of intact FGF23 relative to C-terminal FGF23 in serum. (A) C57BL/6J male mice were fed a diet containing 0.6% inorganic phosphorus (Pi), 1.2% Pi, or 1.65% Pi for 2 weeks. (B) Eight-week-old male mice were fed a diet containing 0.02% or 0.6% inorganic phosphorus (LP) for 2 weeks. (C) Eight-week-old male mice carrying a mutation in the Phex gene (hyp mice) compared to age and sex matched wild-type littermates (control). Data are represented as mean ± SD. All data were analyzed for normality with Shapiro-Wilk test, homogeneity of variance by F test. Samples were in normal distribution and equal variance. One-way ANOVA with Dunnett’s multiple comparison test was performed (A), or unpaired t test was performed compared to WT (B, C). *P <0.05, ****P <0.0001 compared to CONT (control diet). (PDF) [file pone.0315228.s002.pdf]

# Supplementary Figure 2.

## A Duodenum

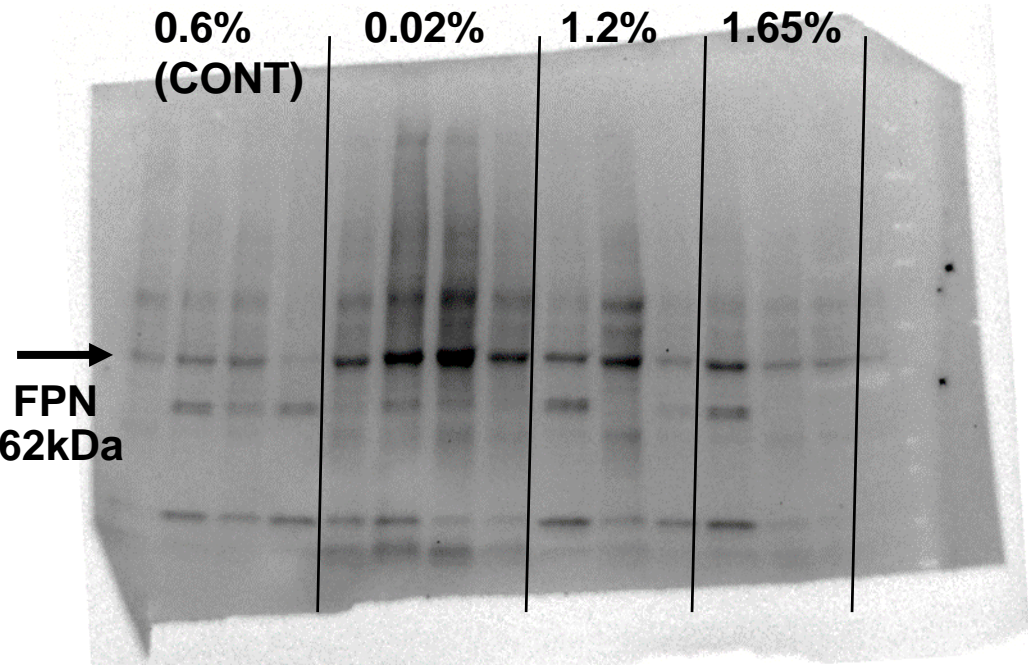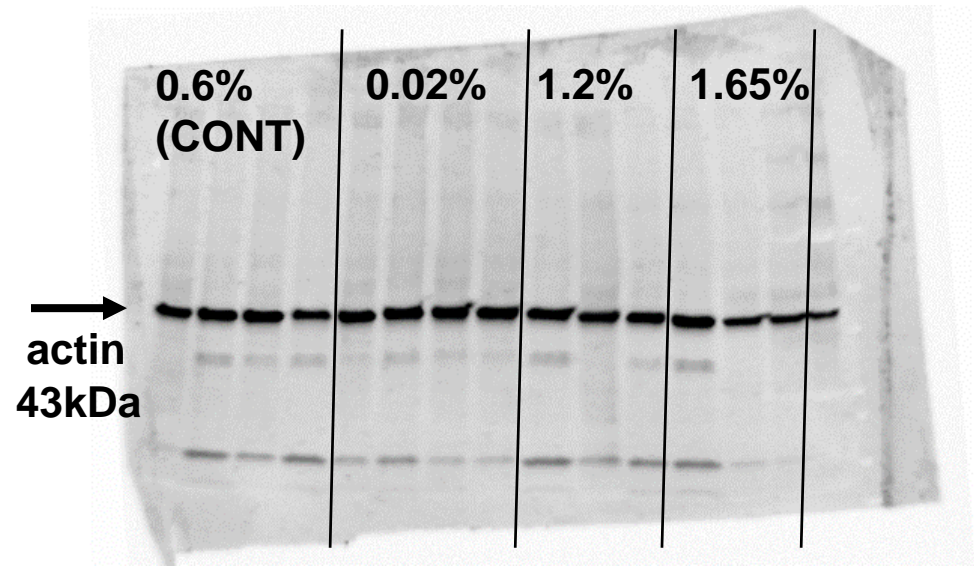

## B Spleen

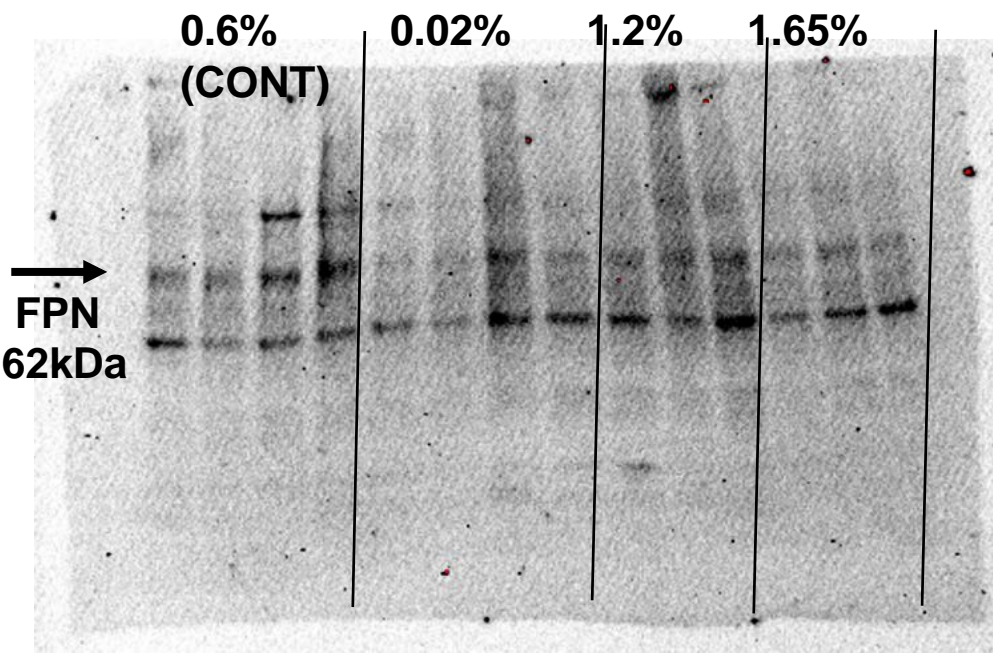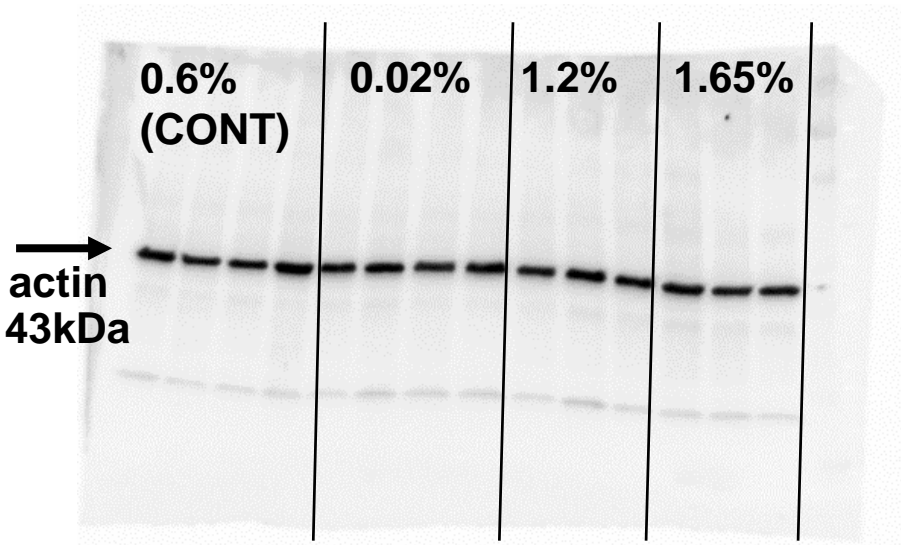

Supplement: S2 Fig — Original uncropped Western Blot images of protein expression of Ferroportin (FPN) and actin in (A) duodenum and (B) spleen. (PDF) [file pone.0315228.s003.pdf]

# Supplementary Figure 3.

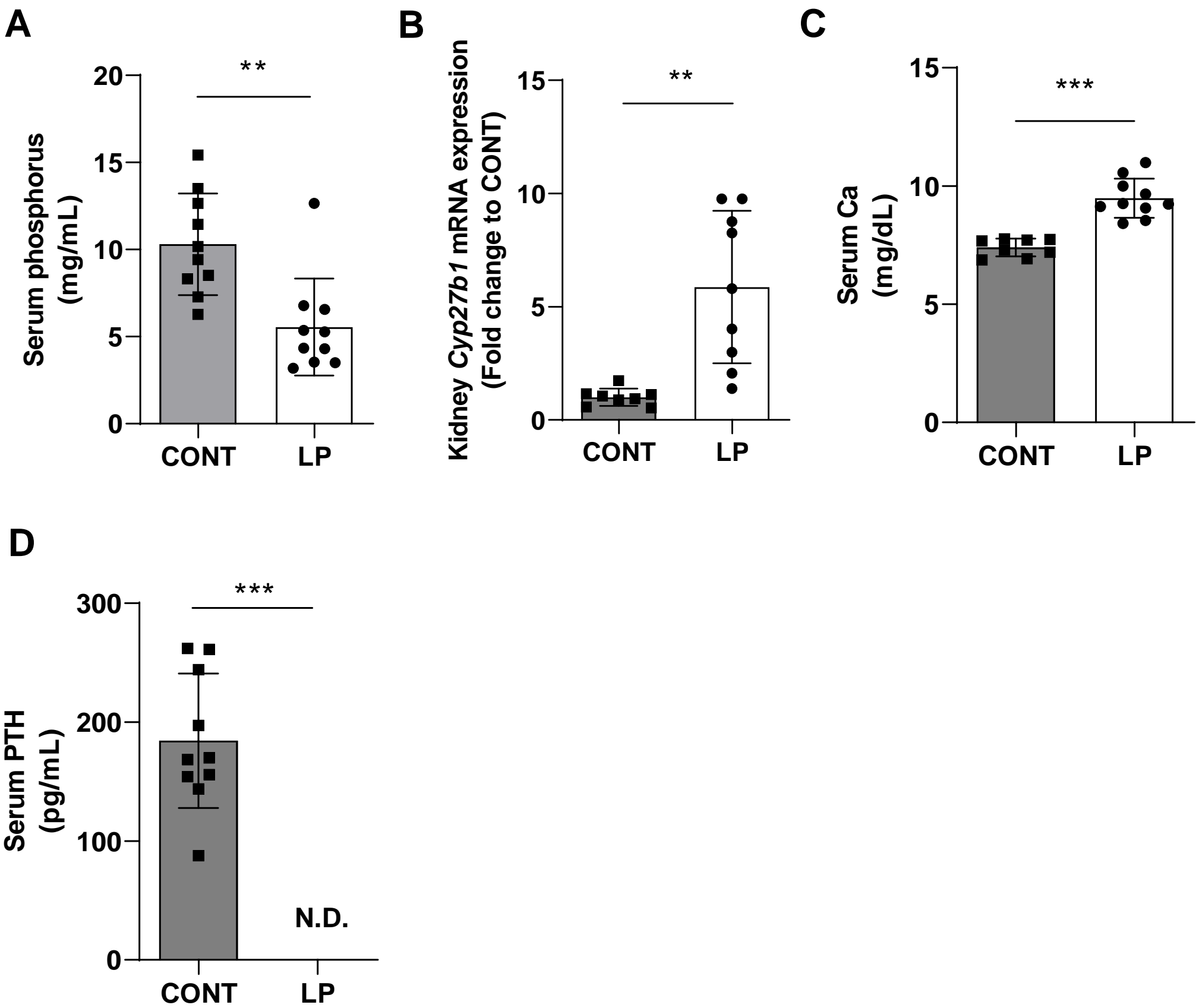

Supplement: S3 Fig — Eight-week old C57BL/6J male mice were fed a diet containing 0.02% inorganic phosphorus (LP) for 2 weeks and compared to age matched C57BL/6J male mice fed normal phosphorus diet (0.6% Pi; CONT). Serum and tissue samples were collected at the end of the experiment. (A) Serum phosphorus levels, (B) Quantitative real-time RT-PCR for renal Cyp27b1 expression. Data are expressed as fold change (2-ΔΔCt) relative to housekeeping gene Hprt. (C) Serum calcium levels, and (D) Circulating PTH levels measured by ELISA. All data were analyzed for normality with Shapiro-Wilk test, homogeneity of variance by F test, and unpaired t test was performed. For samples with normal distribution and equal variances, unpaired t test was performed compared to WT (A). When the samples were in normal distribution but not in homogeneity of variance, the data were analyzed by Welch’s t test (B, C, D). *P < 0.05, **P < 0.01, ***P <0.001 compared to CONT (control diet). (PDF) [file pone.0315228.s004.pdf]

# Supplementary Figure 4.

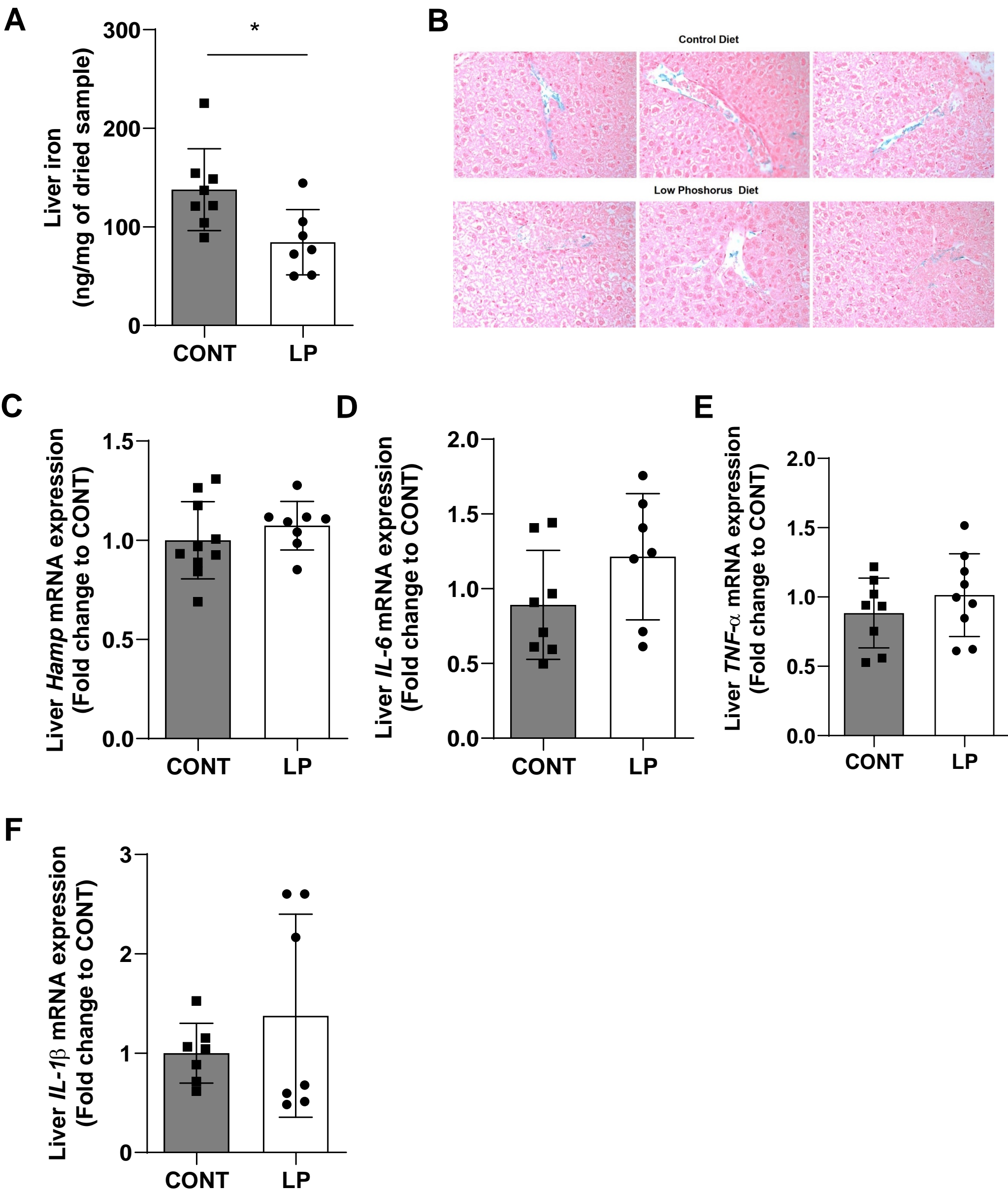

Supplement: S4 Fig — Eight-week old C57BL/6J male mice were fed a diet containing 0.02% inorganic phosphorus (LP) for 2 weeks and compared to age matched C57BL/6J male mice fed normal phosphorus diet (0.6% Pi; CONT). Liver samples were collected at the end of the experiment. (A) Iron content in the liver assessed by the ferrozine colorimetric assay, (B) Representative images of Prussian blue staining for liver iron accumulation (Blue, iron; pink, hepatic nuclei and cytoplasm. (C-F) Quantitative real-time RT-PCR for hepatic inflammatory marker expression. Data are expressed as fold change (2-ΔΔCt) relative to housekeeping gene Hprt. (C) Hepcidin, (D) IL-6, (E) TNF-α, and (F) IL-1β. For samples with normal distribution and equal variances, unpaired t test was performed compared to WT (A, C, D, E). When the samples were in normal distribution but not in homogeneity of variance, the data were analyzed by Welch’s t test (F). *P <0.05 compared to CONT (control diet). (PDF) [file pone.0315228.s005.pdf]
